# Supplementary material for: Magnetosensitivity of tightly bound radical pairs in cryptochrome is enabled by the quantum Zeno effect
Source: Nat Commun. 2024 Dec 30;15:10823. doi: 10.1038/s41467-024-55124-x (PMC11686217; doi:10.1038/s41467-024-55124-x)
Supplement: Supplementary file 1 — Supplementary Information [file 41467_2024_55124_MOESM1_ESM.pdf]

Supplementary Information to "Magnetosensitivity of Tightly Bound Radical Pairs in Cryptochrome is Enabled by the Quantum Zeno Effect"

Matt C. J. Denton, Luke D. Smith, Wenhao Xu, Jodeci Pugsley, Amelia Toghill, Daniel R. Kattnig
Department of Physics and Living Systems Institute
University of Exeter, Stocker Road, Exeter EX4 4QD, U.K.

November 22, 2024

Suppl. Note 1: Investigating Relative Radical Pair Position

To ascertain the variability of compass sensitivity when changing the relative orientation of the two radicals (r), we calculated it over a regular grid of azimuthal and polar angles θ and ϕ, using a strongly asymmetrical pair of recombination rates ($k_S = 10^{3.7} \simeq 5000\,\mu\text{s}^{-1}$, $k_T = 1\,\mu\text{s}^{-1}$) in order to model this behaviour in the quantum Zeno regime. Suppl. Fig. 1 shows that percentage compass sensitivity %S is maximised for $\theta = \frac{\pi}{2}$, hence where r is perpendicular to the dominant hyperfine axis.

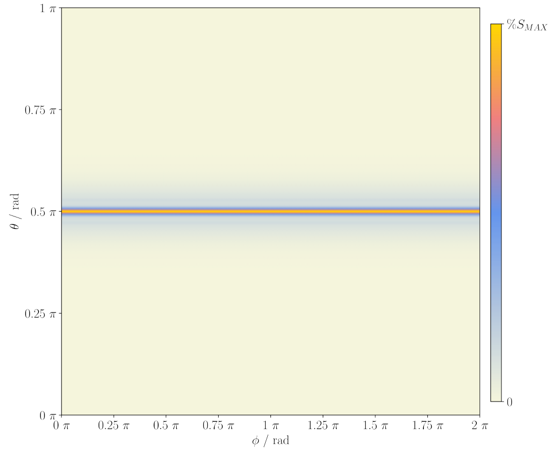

Suppl. Fig. 1: Plot of compass sensitivity as a function of the relative orientation of the radicals r, here achieved through varying angles θ and ϕ. A band of high sensitivity is situated around the line for which $\theta = \frac{\pi}{2}$.

Suppl. Note 2: Molecular Dynamics Trajectory 2

Simulations were also undertaken for MD trajectory 2, which *a priori* promises reduced magnetosensitivity due to an average position of the superoxide well below the $FADH^{\bullet}$ isoalloxazine plane (this is visualised in Suppl. Fig. 2a). Suppl. Fig. 3 summarises our results for the various model calculations. Models 2, 3 and 4 are as described in the main text, representing increasingly complex models of the system. In place of a simulation using model 1, we include a model henceforth referred to as model 2', which includes cross-relaxation terms whilst still using the point-point dipole approximation (as with model 2), yet uses the slightly misaligned N5 hyperfine tensor as used in model 4. Suboptimal placement of superoxide in the model leads to comparatively lower peak MFEs when compared to the first trajectory. Specifically, we observe a maximal effect of merely 0.73% if cross-relaxation terms are fully included (model 2, shown in Suppl. Fig. 3a), which is lower by an order of magnitude compared to Trajectory 1 under analogous conditions. Accounting for the delocalisation of the spin density for the EED tensor (model 3, shown in Suppl. Fig. 3b) has only a small effect; the MFE is bound by 0.6% in this scenario. Remarkably, however, when considering the true orientation of the N5 hyperfine interaction principal axes a strong boost of maximal sensitivity is realised, with MFEs on the order of 1% ($\sim$1.8% for model 2', point-dipole EED shown in Suppl. Fig. 3c; $\sim$1.2% for model 4, the more elaborate treatment shown in Suppl. Fig. 3d). As previously, these large effects are realised for fast singlet recombination rate, hence within the regime characterised by the Zeno effect.

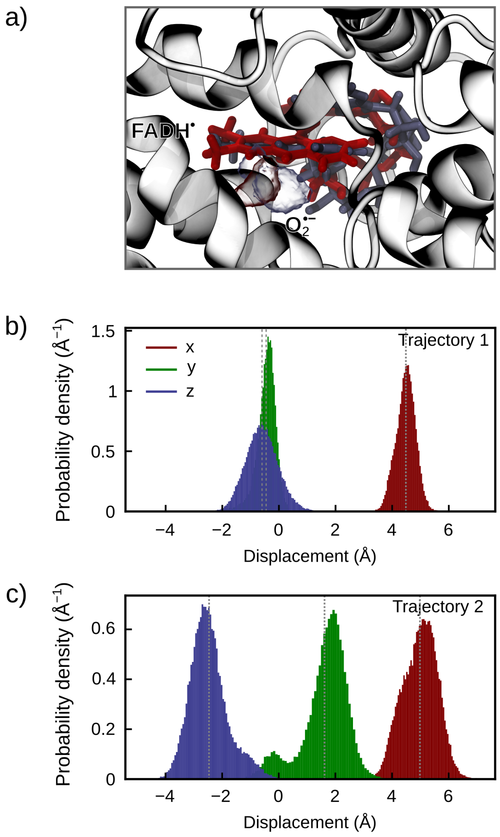

Suppl. Fig. 2: Visualisation of the second molecular dynamics trajectory investigated. It is visualised in frame a, where the $O_2^{\bullet-}$ is no longer in line with the FAD isoalloxazine plane, and now faces the Arg356 residue. Frames b and c show the distance distribution of the two different trajectories. Dotted lines in figures b and c indicate mean values of each distribution.

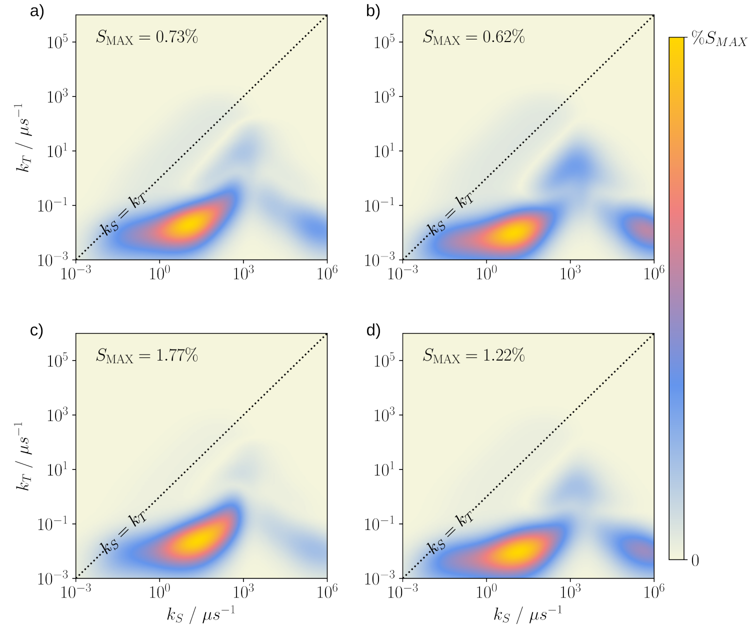

Suppl. Fig. 3: Heatmaps produced from the data from the second molecular dynamics trajectory, in which the alignment of the $O_2^{\bullet -}$ is less optimal than in Trajectory 1. Panels a-d depict, in order, model 2, model 3, model 2' and model 4. Clearly, considering the most accurate representation of the N5 hyperfine coupling tensor, as in panels c and d, there is a boost in sensitivity of an order of magnitude. Comparing panels a with b and c with d, we observe a small decrease in maximum MFE when changing from a point-point approximation for dipolar coupling to one considering the true N5 dipolar coupling tensor.

Suppl. Note 3: Multi-spin Simulations

In order to test the robustness of the model to increasing biologically realistic complexity, we have studied the consequences of increasing the number of hyperfine coupled nuclear spins in the FADH$^\bullet$ radical. Here we considered yet again an idealised system in the point-dipole limit. The superoxide was assumed to be displaced along the molecular x-axis of FADH$^\bullet$ (i.e. along the N5-N10 vector) to a distance of 4.56Å. Due to the substantial computational efforts involved with simulating large spin systems, we further restricted ourselves to the closed system scenario, for which system-environment couplings and the associated relaxation processes are excluded. This configuration thus resembled the idealised systems studied in 3a in the main text. Starting from N5, we included one additional nuclear spin at a time, evaluating the directional MFE as a function of k_S and k_T. Nuclei were added in descending order of their mean hyperfine interaction magnitude, leading to the sequence N5, H5, N10, H6, Hb2. Suppl. Fig. 4 shows the corresponding heatmaps of the relative MFEs for up to 5 nuclear spins, as well as a comparative bar plot showing the peak sensitivity for each system. It is obvious that the peak % magnetosensitivity declines when including increasing numbers of spins. Starting out at 8% for an isolated N5, the inclusion of N10 is associated with a decrease to 5%. Addition of further nuclei gives an effect a little below 1%. While the size of the effect is variable, the shape of the plots remained similar, i.e. asymmetric recombination is most fruitful and large magnetosensitivity is still realised along a triangular region, as was found for the simplest systems studied. We also undertook multi-spin simulations using the hyperfine coupling tensors formed by transforming the existing tensors to a basis that diagonalises N5. The results of this are shown in Suppl. Fig. 5. The remarkable of 48% sensitivity obtained from the single N5 simulation rapidly decreases for 2 spins, before steadying and reaching the same order of magnitude for 5 spins as in the prior case shown in Suppl. Fig. 4. Therefore, this investigation also indicates robustness of a functioning compass, despite a considerable decline in sensitivity form the single spin simulation.

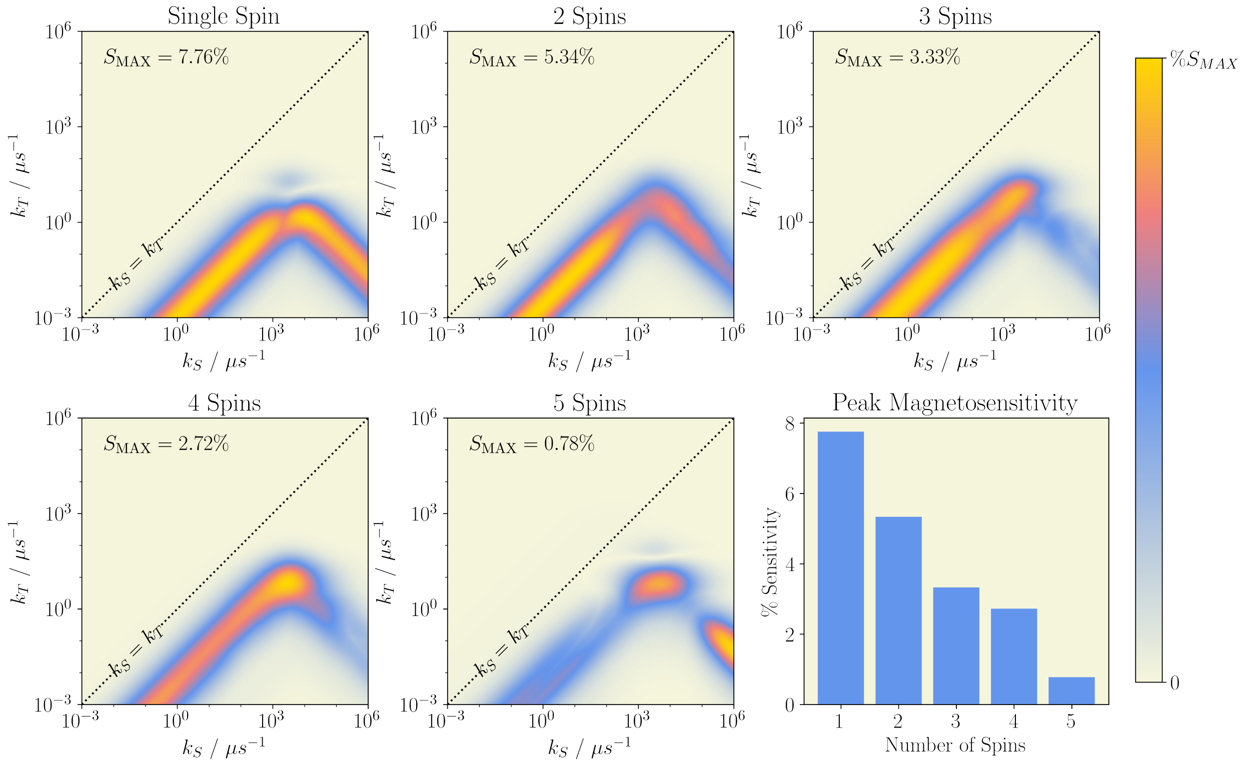

Suppl. Fig. 4: Heatmaps of % magnetosensitivity for a RP system where $H_1 = 0$ (system without system-environment coupling), calculated for increasing numbers of nuclear spins up to five. The data across the plots is normalised for ease of comparison, whilst the peak sensitivity in each heatmap is indicated on each plot by %S$_{MAX}$ as well as in the bar graph. The full hyperfine coupling tensors were used to model the nuclear spins.

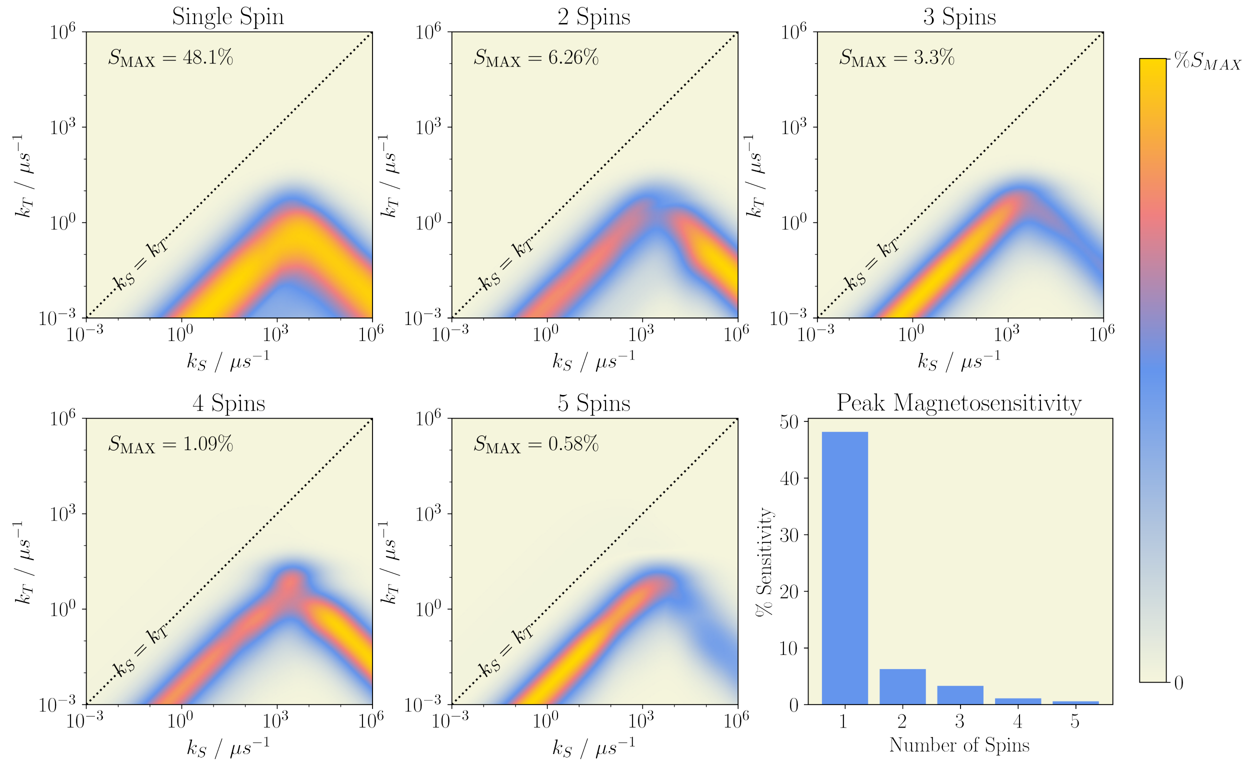

Suppl. Fig. 5: Heatmaps of % magnetosensitivity for a RP system where $H_1 = 0$ (system without system-environment coupling), calculated for increasing numbers of nuclear spins up to five. The data across the plots is normalised for ease of comparison, whilst the peak sensitivity in each heatmap is indicated on each plot by $\%S_{MAX}$ as well as in the bar graph. Simplified hyperfine coupling tensors have been used, transformed to a basis that diagonalises the tensor representing N5.

Suppl. Note 4: Origins of MFEs & Eigenvalue Analysis

The physics underlying the MFE difference for the case in which the magnetic field is oriented in the z-direction compared to the cases in the y and z directions is as follows: The associated eigenvalues, $\lambda^{(z)}$ of the effective Hamiltonian for the case where the magnetic field is aligned in the z-direction, in general and for the relevant parameter regime ($d \gg a, \omega$), are given by:

$$\lambda_S^{(z)} = -\frac{1}{4}\left(\sqrt{4a^2 + \mathrm{i}\kappa + (d - \mathrm{i}\kappa)^2} - d\right) \approx -\frac{1}{2}\left(\mathrm{i}\kappa - \frac{a^2}{d - \mathrm{i}\kappa}\right) \tag{1}$$

$$\lambda_{T_0}^{(z)} = -\frac{1}{4}\left(\sqrt{4a^2 + \mathrm{i}\kappa - (d - \mathrm{i}\kappa)^2} - d\right) \approx -\frac{1}{2}\left(d + \frac{a^2}{d - \mathrm{i}\kappa}\right), \tag{2}$$

where subscripts S and T_0 label the states that are predominantly composed of $|S\rangle$ and $|T_0\rangle$ with a minor contribution of the other. The imaginary parts of λ describe the state decay due to singlet recombination with rate constant κ. While the "$|S\rangle$" state's decay is, apart from a negligible correction given by $\mathrm{Im}\left[\lambda_S^{(z)}\right] \approx -\frac{\mathrm{i}\kappa}{2}$, as expected, the "$|T_0\rangle$" shows the Zeno scaling insofar as the effective decay rate, $\mathrm{Im}\left[\lambda_{T_0}^{(z)}\right]$ decreases with increasing κ for sufficiently large rate constant $\kappa > d$; the asymptotic scaling is $\sim \kappa^{-1}$, which is a characteristic of the quantum Zeno effect. As the relevant block is 2×2, it is straightforward to evaluate the recombination yield associated with nuclear spin projection m_I. Solving for the Laplace-transformed density operator with Laplace parameter $s = 0$, we obtain

$$\phi_s(m_I) = \frac{1}{3}\frac{a^2 k_S (k_S + k_T)}{a^2(k_S + k_T)^2 + k_S k_T\left(d^2 + (k_S + k_T)^2\right)}. \tag{3}$$

Assuming that k_T is negligible next to k_S, i.e. in the quantum Zeno-regime ($\kappa \approx k_S$), this expressions simplifies to

$$\phi_s(m_i) = \frac{1}{3}\frac{a^2 k_S}{k_S\left(a^2 + k_T k_S\right) + d^2 k_T}. \tag{4}$$

The total recombination yield is the average of the yields for the different m_I-components. For non-zero a, Eq. 4 peaks for $k_S = d$.

As is laid out below, second order non-Hermitian perturbation theory can be used to derive the following expressions for the eigenvalues of the four states. To this end, we diagonalise the block degenerate in zeroth order and introduce $|T_\Lambda\rangle = \frac{1}{\sqrt{2}}\left(|T_\Sigma\rangle + |T_0\rangle\right)$ and $|T_\Gamma\rangle = \frac{1}{\sqrt{2}}\left(|T_\Sigma\rangle - |T_0\rangle\right)$ as symmetry adapted zeroth-order states. Using again the zero-order states to label the perturbed states, the eigenvalues for $d \gg a, \omega$ are now given by the following expressions:

$$\lambda_S^{(x)} = -\frac{\mathrm{i}\kappa}{2} + \frac{a^2(d - i\kappa)}{2\left(-4\omega^2 + (d - \mathrm{i}\kappa)^2\right)} \tag{5}$$

$$\lambda_{T_0}^{(x)} = d + \frac{3a^2 d}{18d^2 - 8\omega^2} \tag{6}$$

$$\lambda_{T_\Lambda}^{(x)} = -\frac{d}{2} + \omega - \frac{1}{4}a^2\left(\frac{1}{3d - 2\omega} + \frac{1}{-2\omega + (d - \mathrm{i}\kappa)}\right) \tag{7}$$

$$\lambda_{T_\Gamma}^{(x)} = -\frac{d}{2} - \omega - \frac{1}{4}a^2\left(-\frac{1}{2\omega + 3d} + \frac{1}{2\omega + (d - \mathrm{i}\kappa)}\right). \tag{8}$$

Observe that both the T_Λ and T_Γ-like states show a pronounced quantum Zeno scaling with

$$\mathrm{Im}\left[\lambda_{T_\Lambda, T_\Gamma}^{(x)}\right] = -\frac{a^2\kappa}{4\left((d \mp 2\omega)^2 + \delta k^2\right)} \approx -\frac{a^2\kappa}{4\left(d^2 + \kappa^2\right)}. \tag{9}$$

Different from the situation with the magnetic field along the z-direction, we here end up with two, instead of one, state that depopulates at a rate determined by the quantum Zeno effect, besides the S-like state that is quickly depopulated in both cases. It is this contrast that ultimately gives rise to the large directional MFEs observed for this system. 6 in the main text illustrates the dependence on the imaginary part of the eigenvalues as derived from the perturbation expressions as a function of κ. It is apparent that the resulting dependence directly mirrors the triangular pattern seen in the MFEs. This is so because maximal magnetosensitivity results if the triplet recombination rate is

comparable to the effective depopulation rates as contained in the eigenvalues (which here in part deviate significantly from the rate constant k_S), as a balanced scenario of effective singlet and triplet recombination maximises the impact of changes in the coherent evolution/reaction dynamics. Using computer algebra systems, an analytical expression for the recombination yield for this field direction can in principle be obtained as above. However, it is too intricate to be of practical use. Instead, we will derive an approximate expression. This can be obtained by assuming that T_Δ is not contributing to the magnetic field sensitive dynamics as a result of its relatively large energy gap from the other states. Thus, equating all coherences involving T_Δ to zero, the singlet recombination yield can be evaluated on the basis of the three remaining levels. The result is impractically complicated: a simplified, but approximate result can be obtained by expanding the numerator and denominator of the rational function in k_T and ω, retaining terms up to second order, and neglecting k_T next to k_S in the resulting expression:

$$\phi_s(m_i) = \frac{a^6 k_T + 4a^4 k_S\left(2\omega^2 + \mathrm{k}_0^2\right) + 16a^2\omega^2 k_T\left(d^2 + \delta k^2\right)}{3\left(a^6 k_T k_S + a^4\left(4\omega^2 k_S^2 + \mathrm{k}_0^2\left(d^2 + 5k_S^2\right)\right) + 16a^2\omega^2 k_T k_S\left(d^2 + k_S^2\right) + 16\omega^2 \mathrm{k}_0^2\left(d^2 + k_S^2\right)^2\right)}. \tag{10}$$

The difference between Eq. 3 and Eq. 10 provides a measure of the anisotropy of the MFE. For the parameter ranges used here the approximation closely matches the full calculation.

Finally, for the magnetic field along the y-direction a scenario similar to that for the z-direction follows in the light of the perturbation treatment. As observed for the z-direction, the imaginary part of the eigenvalue of one state is found to show a marked Zeno-type dependence. The recombination field is thus comparable to that for the z-field and again smaller than for x-field direction.

Suppl. Note 5: Additional Relaxation Models

Suppl. Fig. 6 below adds to the plots shown in the main text showing the MFEs extracted from the system when under the influence of two further interaction Hamiltonians: the case involving the inclusion of the exchange interaction (EXR) for varying $\langle X_i X_j \rangle \tau_C$, and the case involving relaxation originating from random field noise (RFR). This shows the detrimental effect of RFR to this system, as well as the fact that including the EED interaction along with it does not recover MFEs. The system is considerably more resilient, however, to the inclusion of the fluctuating exchange interaction.

Furthermore, Suppl. Fig. 7 shows heatmaps of a system where singlet-triplet dephasing (STD) has been directly included to elucidate the effect. As seen above for the case involving fluctuating EXR (incidentally also a source of STD), we see no appreciable decline in magnetic field sensitivity, but a broadening of the range at which this maximal effect occurs. Most notably, this region broadens to include ranges where less asymmetrical recombination (fast k_S) is required. This indicates that STD may serve as a de-facto substitute for recombination.

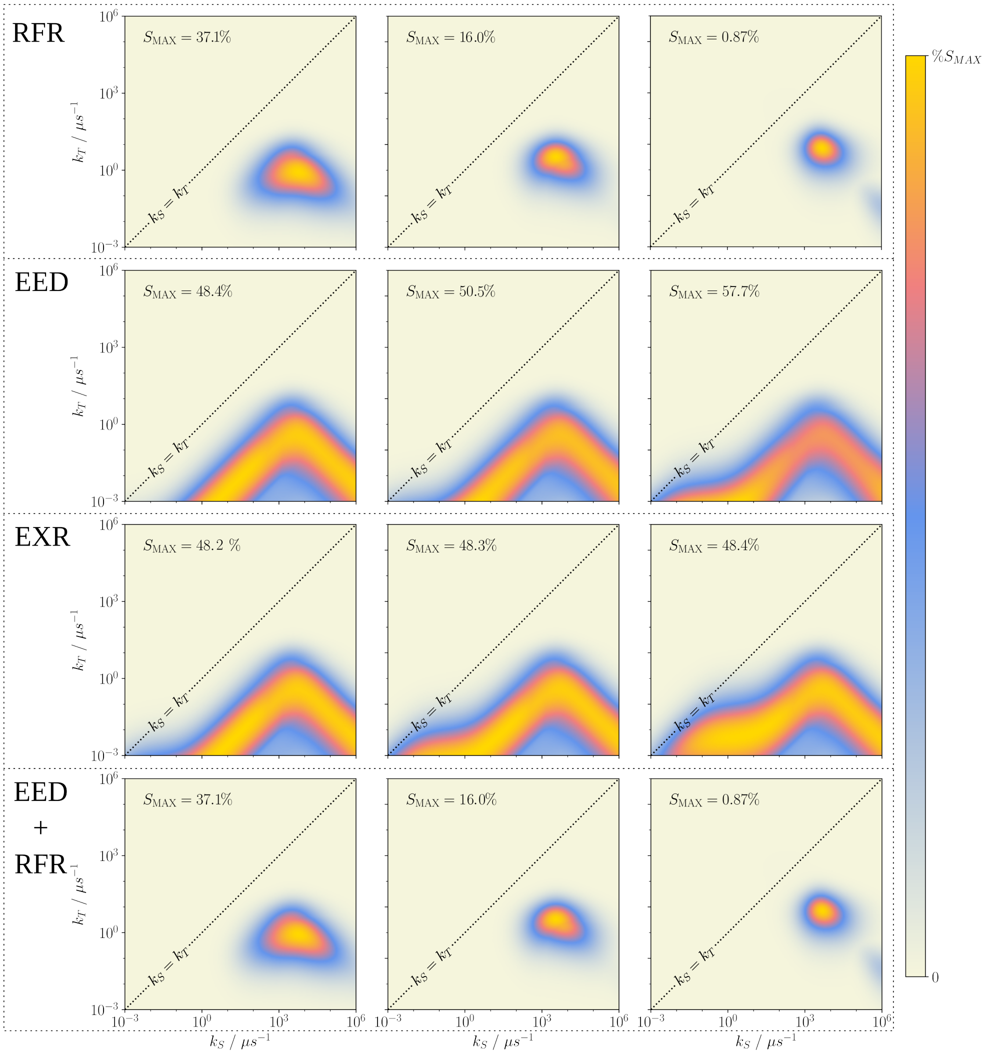

Suppl. Fig. 6: Heatmaps of % magnetosensitivity for 4 different environmental interactions (RFR, EED, and EXR correspond to random field relaxation, electron-electron dipolar coupling, and exchange interaction relaxation) for $\langle X_i X_j \rangle \tau_C = 0.1\ \mu\text{s}^{-1}$, $1\,\mu\text{s}^{-1}$, and $10\,\mu\text{s}^{-1}$ (columns left to right), where $\langle X_i X_j \rangle \tau_C$ serves as an analogue for coupling strength. The singlet yield has been sampled over 250 possible magnetic field orientations to evaluate % sensitivity over a logarithmic scale of singlet and triplet recombination rates k_S and k_T. For ease of presentation, each heatmap has been self normalised, but the peak sensitivity value (%S_{MAX})for each is labelled on each plot. Peaks in sensitivity are most resilient to increased coupling strength when the system is subject to dipolar interactions, whereas random field relaxation is very detrimental to magnetosensitivity. Combining RFR with dipolar coupling cannot salvage peaks in sensitivity, suggesting dominance of RFR when these two interactions are combined. The central diagonal is indicated here to show the line of symmetric recombination $k_S = k_T$

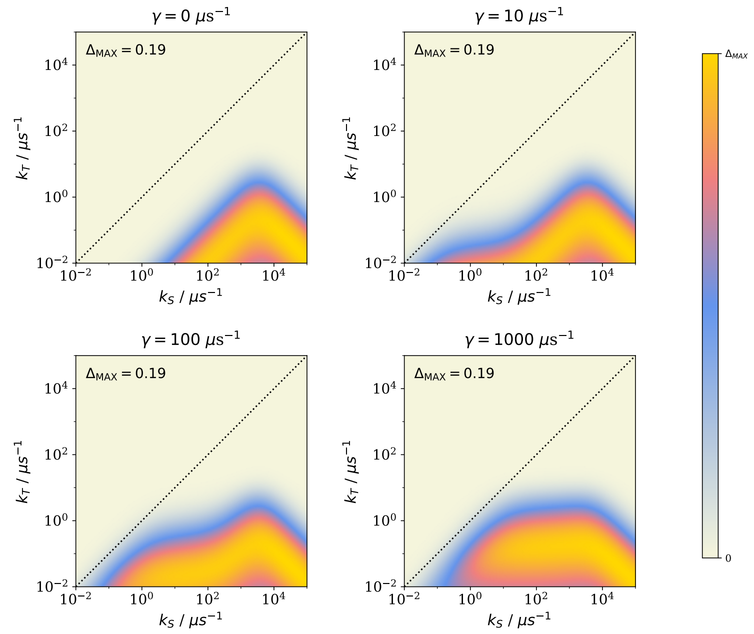

Suppl. Fig. 7: Heatmaps showing simulations for systems where singlet-triplet dephasing has been included at varying rates. Increasing the dephasing rate to $\gamma = 1000 \mu s^{-1}$, a broadening of the peak sensitivity range, in the direction of slower k_S, illustrates that singlet-triplet dephasing may function as a substitute for recombination if it itself is slower.

Suppl. Note 6: Evaluating Coherence and Entanglement

We evaluated coherence and entanglement measures for the RP system. The l_1 norm coherence of the RP is defined as:

$$C_{l1}(\hat{\rho}) = \sum_{i \neq j} |\hat{\rho}_{ij}|. \tag{11}$$

This was evaluated for both electron coherence as well as total coherence in the magnetic field orientations associated with minimal and maximal singlet yield. Total coherence has been previously shown as having a direct correlation to compass sensitivity [1, 2]. Suppl. Fig. 8A and B show mean coherence and absolute coherence difference respectively plotted against absolute difference in singlet yield $\Delta\Phi_S$, varying $\mathbf{r}_{12}$ with changing colour.

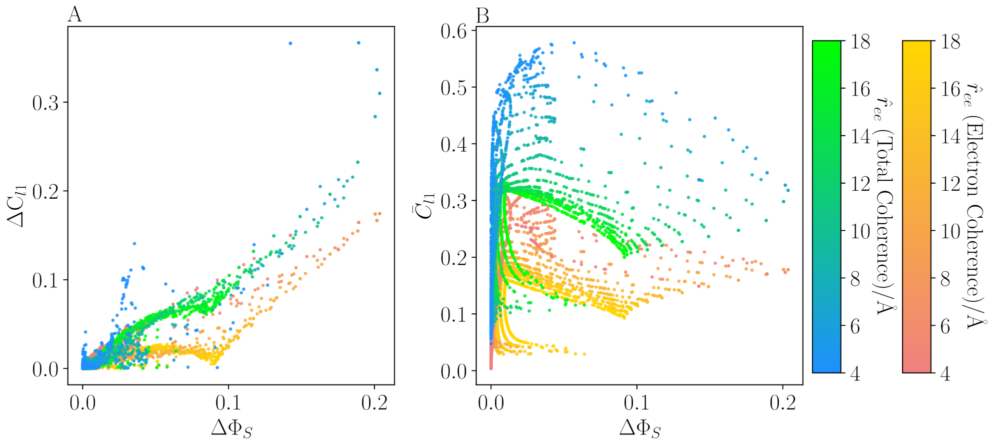

Suppl. Fig. 8: Pair of figures showing l_1 norm coherence C_{l1} against singlet yield difference $\Delta\Phi_S$. Panel A uses the difference in the coherence measure associated with the magnetic field direction of minima and maximal yield, ΔC_{l1}, whereas panel B uses the mean coherence associated with these two directions, $\overline{C}_{l1}$. Panel A indicates a strong positive correlation between ΔC_{l1} and $\Delta\Phi_S$, yet not between the latter and $\overline{C}_{l1}$. This indicates that variability in coherence aids in the establishment of singlet yield anisotropy, and thus compass sensitivity. However, it also shows that increasing mean coherence in the system does not induce higher anisotropy.

Suppl. Fig. 8 indicates a significant correlation between ΔC_{l1} and $\Delta\Phi_S$ (particularly for total coherence). However, there is no strong correlation shown in the $\overline{C}_{l1}$ plot, suggesting that high magnitude of coherence is not the key to extracting high sensitivity, but that variability in coherence is most important.

Negativity was used as a measure for system entanglement, defined as:

$$N(\hat{\rho}) = \frac{||\hat{\rho}^{T_A}||_1 - 1}{2}, \tag{12}$$

where $\hat{\rho}^{T_A}$ is the trace norm of the partial transpose of $\hat{\rho}$ with respect to the subsystem A, defined as $\langle i_A, j_B|\hat{\rho}^{T_A}|m_A, n_B\rangle \equiv \langle m_A, j_B|\hat{\rho}|i_A, n_B\rangle$ [3, 4].

The investigation of negativity shown in Suppl. Fig. 9 shows a strong correlation between ΔN and $\Delta\Phi_S$, indicating that the sensitivity of the RP is also strongly correlated with the entanglement of the RP. Additionally, this trend hold true across the whole range of $\hat{r}$ values. However, as with coherence above, higher mean negativity does not correlate with higher yield variability, as before, indicating that higher ΔN is more important in raising sensitivity.

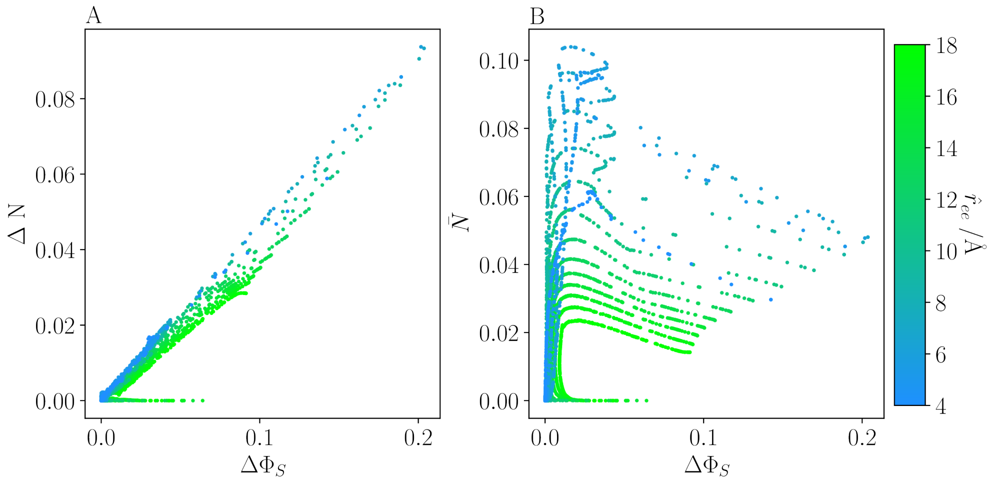

Suppl. Fig. 9: Pair of figures showing two measures of entanglement: difference in negativity ΔN and mean negativity $\overline{N}$, both plotted against singlet yield difference $\Delta\Phi_S$. Panel A and B show absolute difference ΔN and mean negativity $\overline{N}$ respectively. An obvious correlation can be observed between ΔN and $\Delta\Phi_S$, indicating that increasing ΔN is a resource for the compass, establishing greater singlet yield anisotropy. Similar to the coherence measures shown in Suppl. Fig. 8, a greater mean value of negativity ($\overline{N}$) does not increase the singlet yield anisotropy, illustrating that larger entanglement is not directly correlated to increasing compass sensitivity.

Suppl. Note 7: Analysis of Magnetic Field Effect Against Field Strength

Suppl. Fig. 10 shows a plot of MFE ($\chi = \overline{\Phi}(B_0)/\overline{\Phi}(B_0 = 0) - 1$) against field strength, where $\overline{\Phi}$ represents the singlet yield averaged over magnetic field directions, for a choice of asymmetric rates $k_T = 1\mu s^{-1}$ and $k_S = 10^3\mu s^{-1}$ found to be auspicious for our toy model neglecting relaxation. Overall, the effect exhibits a low-field effect-like structure, but with vastly enhanced and broadened low-field response extending into the tens of mT region.

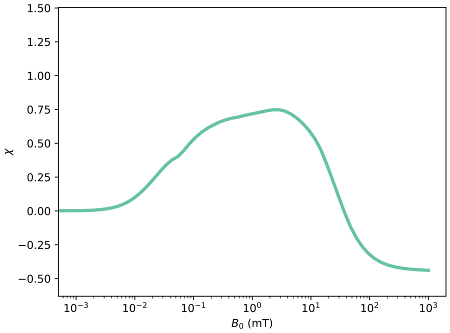

Suppl. Fig. 10: Figure showing χ plotted against field strength for a FAD/Z RP. Rates chosen here are $k_S = 10^3\mu s^{-1}$, $k_T = 1\mu s^{-1}$

Suppl. Note 8: Time Evolution of the Singlet and Triplet Probability

Here, simulations were undertaken to show evolution of singlet and triplet probability over time for a toy model RP over a $2\mu s$ time frame. This is shown in Suppl. Fig. 11. Two orthogonal field directions ($\hat{x}$ and $\hat{z}$) were investigated, as well as including the effect of singlet-triplet dephasing. These demonstrate that, for the $\hat{z}$ direction, the triplet probability decays from 1 to ~0.8 within the first 250 ns but is then "frozen" after that. Contrarily, in the $\hat{x}$ direction, the population continues to decay to under 0.4 by 2 μs resulting in a significant difference in yields.

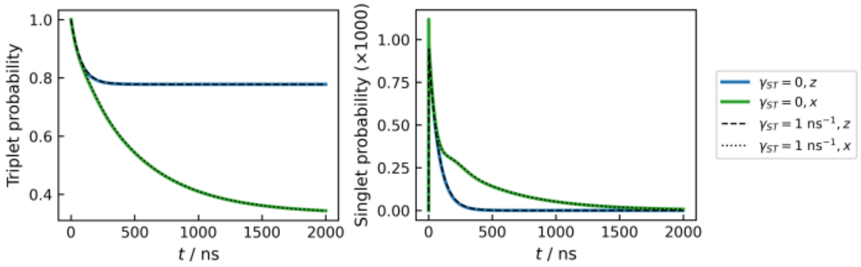

Suppl. Fig. 11: Plots showing the evolution of singlet and triplet probability over a 2μs time frame of a one-nitrogen radical pair with $A_{\|} = 49$ MHz, the second radical displaced by 4.56 Åalong $\hat{x}$, $k_T = 0$, and $k_S = 3000\ \mu s^{-1}$. The triplet probability flatlines at ~0.8 for the case of the $\hat{z}$-aligned field, but decays to under 0.4 for the $\hat{x}$-aligned field. Including singlet-triplet dephasing at a rate of $\gamma = 1 ns^{-1}$ induces minimal deviation in the spin dynamics.

Suppl. Note 9: Singlet-Born Radical Pairs

Prior work has suggested that a RP initialised in the triplet spin state will exhibit greater magnetosensitivity than otherwise [5]. In order to elucidate how changing the initial RP spin state $\hat{\rho}_0$ impacts MFEs, we simulated two (otherwise identical) RPs, intialising one in the singlet spin configuration, and the other in the triplet (as is the case in all other simulations herein). Heatmaps of the maximal difference in singlet yield (Δ_{MAX}) are shown in Suppl. Fig. 12. Clearly, there is a comparatively greater magnetosensitivity realised from a triplet-born RP.

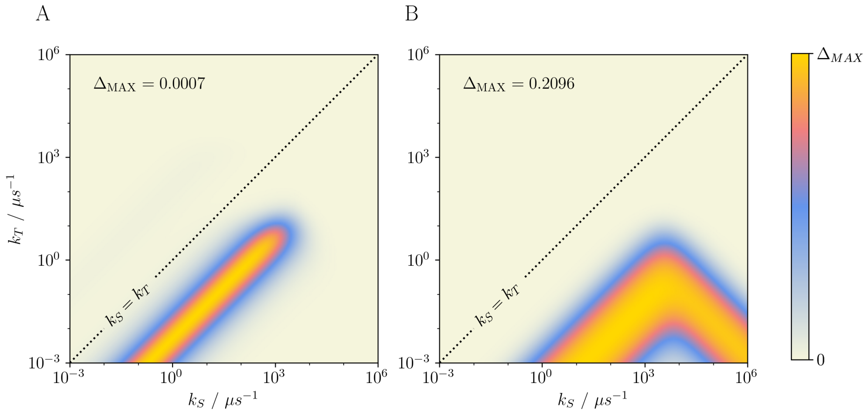

Suppl. Fig. 12: Heatmap plots comparing maximum difference in singlet yield (Δ_{MAX}). Plot A is for a singlet-born RP, whereas plot B is for a triplet-born RP. A clear comparative boost in magnetosensitivity is observed for the triplet-born simulation.

Suppl. Note 10: Kinetics of the Flavin Semiquinone-Superoxide Reaction

The activation of molecular oxygen by flavins and flavoproteins has been reviewed extensively [6, 7]. Typical reaction stages are as follows: Reduced flavins react with O_2 in an electron transfer reaction, which yields a caged radical pair of the superoxide anion and the corresponding flavin semiquinone. After spin inversion, the latter is often found to collapse into the flavin C(4a)-hydroperoxide. Pulse radiolysis measurements suggest that this bimolecular association process is fast, while the subsequently decayed to oxidized flavin and hydrogen peroxide at an O_2 independent rate is rate determining [6]. The reaction rate constant of the fast association reaction is central to the suggested reaction mechanism, but difficult to assess as the available kinetic data are limited to the overall reaction.

Massey [6] obtains a rate constant on the order of $k_2 \sim 10^8$ Lmol^{-1}s^{-1} for the conversion of the flavin semiquinone, FH$^\bullet$, and $O_2^{\bullet-}$ to the oxidized flavin, F, and H_2O_2, in free solution. This diffusion-influenced bimolecular rate constant can be used to establish a lower bound of the pertinent association rate constant of FH$^\bullet$ and $O_2^{\bullet-}$ in the encounter complex, which can serve as an order of magnitude estimate for k_S in the suggested model. Assuming a kinetic scheme comprising the diffusive association of the reactants to a precursor complex (rate constant k_d), its dissociation to the free reactants (rate constant k_{-d}, and its actual reaction (e.g. forming the hydroperoxide; rate constant k_r), a steady state analysis gives $k_2^{-1} = (K_A k_r)^{-1} + k_d^{-1}$, where $K_A = k_d/k_{-d}$ is the association constant [8]. For non-interacting reactants, the diffusion rate constant is given as $k_d = 4\pi D\sigma$, where $\sigma \approx 6.5$ Å is the encounter distance in the reactive complex and D is the mutual diffusion coefficient. Estimating $K_A = 4\pi\sigma^2\delta\sigma$ following Sutin [9], where $\delta\sigma$ is the thickness of the reaction zone, which is frequently postulated to equal 0.8 Å, and D from the Stokes-Einstein-Sutherland relationship, $D = k_B T/(3\pi\eta\sigma)$, with $\eta \approx 1$ mPa s, we obtain $k_r \approx 400\mu$s^{-1}. As the estimate neglects the subsequent, rate-limiting decomposition of the C(4a)-hydroperoxide, this value can serve as a lower bound of the actual association rate constant. Thus, the predicted k_r is of the required order of magnitude to induce large magnetosensitivity.

In flavoproteins the reactivity is clearly modulated by the protein environment and the overall reaction with O_2 may be orders of magnitude faster or slower, depending on the specific flavoprotein and its class. The formation of the hydroperoxide is often postulated; alternative reaction pathways exist, such as the formation of flavin-N5-peroxides or

pathways avoiding the formation of covalent oxygen adducts altogether. A noteworthy example, for which the flavin hydroperoxide is well established, is the reaction of the neutral flavin radical of glucose oxidase and O_2^-, which reacts to the flavin hydroperoxide with a rate constant of $1 \times 10^9 \mathrm{L}^{-1}\mathrm{mol}^{-1} s^{-1}$ [6], suggesting a swift intrinsic association reaction, as required by the suggested mechanism.

The fast addition of superoxide to the flavin semiquinone is also supported by recent density functional calculations of the oxidation reaction in a bacterial luciferase [10, 11]. Stare concludes that the rate-limiting factor of the oxidation is associated with the change in the spin state of the system (which is treated explicitly here). The minimum energy crossing point of the triplet hydroperoxide-flavin semiquinone complex and the singlet oxidation products was found only 3.4 kcal/mol above the reactants, suggesting a fast reaction using typical well frequencies (on the order of $k_B T/h$).

The reoxidation of the reduced or semi-reduced flavin cofactor has been investigated for the plant cryptochrome AtCry1 [12, 13]. The data are in line with the suggested mechanism and the reactivity of flavins as established for free solutions. Specifically, the reaction appears autocatalytic with fully reduced flavin FADH^- oxidizes more rapidly than the $\mathrm{FADH}^\bullet$ radical, in agreement with a reactive $\mathrm{FADH}^\bullet$/superoxide radical pair. The overall reaction rates with O_2 are however small ($\sim 50\ \mathrm{M}^{-1}\ \mathrm{s}^{-1}$ for FADH^- in AtCry1). This reflects the fact that the access of oxygen to the flavin binding pocket is sterically hindered, rather than a low intrinsic reactivity. In fact, the reaction of $\mathrm{FADH}^\bullet$ and superoxide/hydroperoxide in the binding pocket could not be resolved in these experiments but is expected to occur at a much faster rate [14].

In light of the available data and insights from computational models, it is apparent that the singlet reaction rate constants required to elicit the described effect are feasible even in systems that exhibit slow overall kinetics.

Suppl. Note 11: Comparison of Cryptochrome 1 and Cryptochrome 4

The simulations throughout this section were undertaken using model parameters from a Cry4 system. We have used Cry4 as it is the only avian cryptochrome with an associated crystal structure that has been determined by x-ray crystallography, which was necessary for our molecular dynamics simulations. Suppl. Fig. 13 and Suppl. Fig. 14 illustrate that in positions relevant to this mechanism, the variants of cryptochrome show no differences that indicate that there would result any loss of universality in our simulations.

```
                                   361      .         .         .    4         .         .         . 430
1 sp|O77059|CRY1_DROME                FPLIDGAMRQLLAEGWLHHTLRNTVATFLTRGGLWQSWEHGLQHFLKYLLDADWSVCAGNWMWVSSSAFE
2 sp|Q5IZC5|CRY1_ERIRU                FPWIDAIMTQLRQEGWIHHLARHAVACFLTRGDLWISWEEGMKVFEELLLDADWSVNAGSWMWLSCSSFF
3 sp|Q8QG61|CRY1_CHICK                FPWIDAIMTQLRQEGWIHHLARHAVACFLTRGDLWISWEEGMKVFEELLLDADWSVNAGSWMWLSCSSFF
4 sp|Q6ZZY0|CRY1_SYLBO                FPWIDAIMTQLRQEGWIHHLARHAVACFLTRGDLWISWEEGMKVFEELLLDADWSVNAGSWMWLSCSSFF
5 tr|A0A219TAG4|A0A219TAG4_COLLI      FPWIDAIMTQLRQEGWIHHLARHAVACFLTRGDLWISWEEGMKVFEELLLDADYSINAGNWMWLSASAFF
6 tr|A0A2I4SZI9|A0A2I4SZI9_ERIRU      FPWIDAIMTQLRQEGWIHHLARHAVACFLTRGDLWISWEEGMKVFEELLLDADYSINAGNWMWLSASAFF
7 tr|Q9I912|Q9I912_DANRE              FPWIDAIMTQLLQEGWIHHLARHAVACFLTRGDLWISWEEGMKVFEEFLLDADYSVNAGNWMWLSASAFF
8 sp|Q43125|CRY1_ARATH                YPLVDAGMRELWATGWLHDRIRVVVSSFFVK-VLQLPWRWGMKYFWDTLLDADLESDALGWQYITGTLPD
9 tr|M4WL45|M4WL45_PYRAP              FPWIDAIMTQLREEGWIHHLARHAVACFLTRGNLWISWEEGMKVFEELLLDADWSVNAGMWMWLSCSSFF
```

Suppl. Fig. 13: Above are shown multiple sequence alignment of the central part of the sequence of various cryptochromes, namely, from top to bottom, the cryptochrome of *Drosophila melanogaster*; cryptochrome 1 from *Erithacus rubecula*, *Gallus gallus*, and *Sylvia borin*; cryptochrome 4 of *Columba livia*, *Erithacus rubecula*, and *Danio rerio*; cryptochrome 1 from *Arabidopsis thaliana*; and and cryptochrome 2 from *Pyrrhocoris apterus*. The residues putatively surrounding a superoxide molecule bound in the FAD binding pocket and facing N5 are highlighted, whereby green (yellow) indicates agreement with the residue found at the equivalent position in the sequence of cryptochrome 4 from *Columba livia*, the only avian cryptochrome for which the structure has currently been determined by x-ray crystallography and which was used to underpin the molecular dynamics simulations of superoxide bound to cryptochrome. The grey highlights mark the second tryptophan of the tryptophan triad/tetrad. For the key residues, deviations are seen for the cryptochrome from *D. melanogaster* and cryptochrome 1 from *A. thaliana* only, for which the N5-facing Asn is substituted by Cys and Asp, respectively. These substitutions are known to stabilize the semiquinoid FAD in the anionic and protonated form.

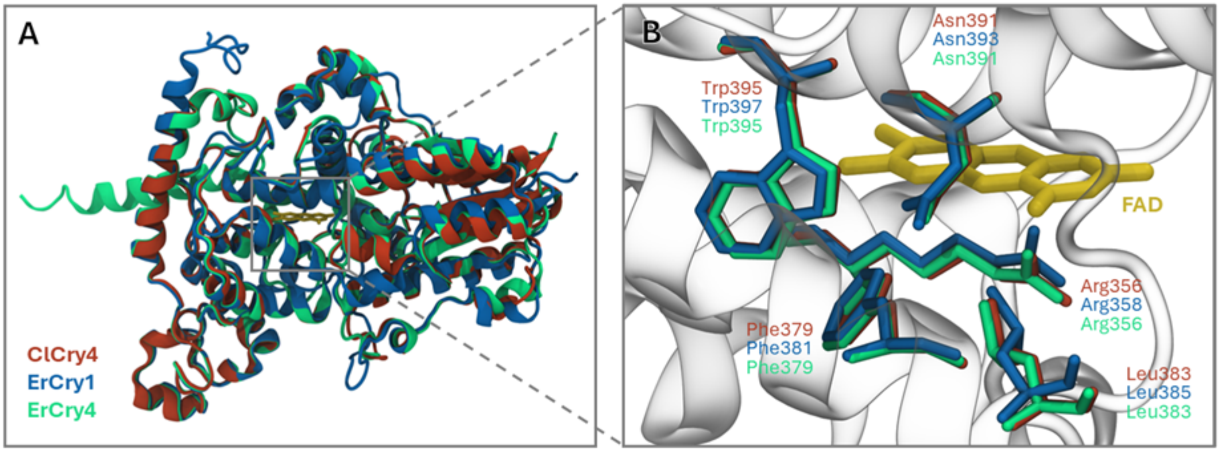

Suppl. Fig. 14: Comparison of the crystal structure of the photolyase homology region of cryptochrome 4 from *Columba livia* (red; ClCry4; PDB ID: 6PU0) and the AlphaFold structure predictions of cryptochrome 1 (blue; ErCry1; UniProt: Q5IZC5) and cryptochrome 4 (green; ErCry4; UniProt: A0A2I4SZI9) from *Erithacus rubecula*: A) overall fold and B) FAD binding pocket highlighting residues that putatively bind superoxide. The fold at the FAD binding site is highly conserved in terms of the overall structure as well as pertinent interaction residues, suggesting that, on the level accessible through mere structure, the proposed model of magnetosensitive re-oxidation applies equally to various cryptochromes, including cryptochrome 1 and cryptochrome 4 from the night migratory European Robin.

Suppl. Note 12: Simulation Parameters

12.1. Trajectory 1

12.1.1. Model 1

$$D_{ee} = \begin{pmatrix} -1109.9 & 0.0 & 0.0 \\ 0 & 554.9 & 0.0 \\ 0 & 0 & 554.9 \end{pmatrix} \text{MHz} \tag{13}$$

$$\sigma^2 = \begin{pmatrix} 65557 & 0 & 0 & 0 & -32778 & 0 & 0 & 0 & -32778 \\ 0 & 0 & 0 & 0 & 0 & 0 & 0 & 0 & 0 \\ 0 & 0 & 0 & 0 & 0 & 0 & 0 & 0 & 0 \\ 0 & 0 & 0 & 0 & 0 & 0 & 0 & 0 & 0 \\ -32778 & 0 & 0 & 0 & 16389 & 0 & 0 & 0 & 16389 \\ 0 & 0 & 0 & 0 & 0 & 0 & 0 & 0 & 0 \\ 0 & 0 & 0 & 0 & 0 & 0 & 0 & 0 & 0 \\ 0 & 0 & 0 & 0 & 0 & 0 & 0 & 0 & 0 \\ -32778 & 0 & 0 & 0 & 16389 & 0 & 0 & 0 & 16389 \end{pmatrix} \text{MHz}^2 \tag{14}$$

12.1.2. Model 2

$$D_{ee} = \begin{pmatrix} -1031.9 & 143.1 & 215.8 \\ 143.1 & 534.6 & -22.8 \\ 215.8 & -22.8 & 497.4 \end{pmatrix} \text{MHz} \tag{15}$$

$$\sigma^2 = \begin{pmatrix} 61292 & 5938 & -6807 & 5938 & -32556 & -1878 & -6807 & -1878 & -28736 \\ 5938 & 8462 & 3327 & 8462 & -3429 & -1780 & 3327 & -1780 & -2509 \\ -6807 & 3327 & 49727 & 3327 & 9924 & -4155 & 49727 & -4155 & -3117 \\ 5938 & 8462 & 3327 & 8462 & -3429 & -1780 & 3327 & -1780 & -2509 \\ -32556 & -3429 & 9924 & -3429 & 18680 & 528 & 9924 & 528 & 13876 \\ -1878 & -1780 & -4155 & -1780 & 528 & 871 & -4155 & 871 & 1351 \\ -6807 & 3327 & 49727 & 3327 & 9924 & -4155 & 49727 & -4155 & -3117 \\ -1878 & -1780 & -4155 & -1780 & 528 & 871 & -4155 & 871 & 1351 \\ -28736 & -2509 & -3117 & -2509 & 13876 & 1351 & -3117 & 1351 & 14860 \end{pmatrix} \text{MHz}^2 \tag{16}$$

12.1.3. Model 3/4

$$D_{ee} = \begin{pmatrix} -821.7 & 441.7 & 198.0 \\ 441.7 & 289.0 & -67.3 \\ 198.0 & -67.3 & 532.7 \end{pmatrix} \text{MHz} \tag{17}$$

$$\sigma^2 = \begin{pmatrix} 48642 & -12059 & 1840 & -12059 & -19181 & -2963 & 1840 & -2963 & -29460 \\ -12059 & 6825 & 3078 & 6825 & 3990 & -993 & 3078 & -993 & 8069 \\ 1840 & 3078 & 49461 & 3078 & 3659 & -16975 & 49461 & -16975 & -5499 \\ -12059 & 6825 & 3078 & 6825 & 3990 & -993 & 3078 & -993 & 8069 \\ -19181 & 3990 & 3659 & 3990 & 8929 & -181 & 3659 & -181 & 10253 \\ -2963 & -993 & -16975 & -993 & -181 & 6232 & -16975 & 6232 & 3144 \\ 1840 & 3078 & 49461 & 3078 & 3659 & -16975 & 49461 & -16975 & -5499 \\ -2963 & -993 & -16975 & -993 & -181 & 6232 & -16975 & 6232 & 3144 \\ -29460 & 8069 & -5499 & 8069 & 10253 & 3144 & -5499 & 3144 & 19208 \end{pmatrix} \text{MHz}^2 \tag{18}$$

12.2. Trajectory 2

12.2.1. Model 2/2'

$$D_{ee} = \begin{pmatrix} -349.2 & -153.8 & 286.2 \\ -153.8 & 216.2 & 83.8 \\ 286.2 & 83.8 & 132.9 \end{pmatrix} \text{MHz} \tag{19}$$

$$\sigma^2 = \begin{pmatrix} 73739 & -18165 & -17796 & -18165 & -39174 & 9174 & -17796 & 9174 & -34565 \\ -18165 & 11548 & 2767 & 11548 & 10007 & -5287 & 2767 & -5287 & 8158 \\ -17796 & 2767 & 15629 & 2767 & 13517 & 128 & 15629 & 128 & 4279 \\ -18165 & 11548 & 2767 & 11548 & 10007 & -5287 & 2767 & -5287 & 8158 \\ -39174 & 10007 & 13517 & 10007 & 22869 & -4356 & 13517 & -4356 & 16305 \\ 9174 & -5287 & 128 & -5287 & -4356 & 3287 & 128 & 3287 & -4818 \\ -17796 & 2767 & 15629 & 2767 & 13517 & 128 & 15629 & 128 & 4279 \\ 9174 & -5287 & 128 & -5287 & -4356 & 3287 & 128 & 3287 & -4818 \\ -34565 & 8158 & 4279 & 8158 & 16305 & -4818 & 4279 & -4818 & 18261 \end{pmatrix} \text{MHz}^2 \tag{20}$$

12.2.2. Model 3/4

$$D_{ee} = \begin{pmatrix} -356.6 & -14.2 & 382.1 \\ -14.2 & 257.8 & 8.0 \\ 382.1 & 8.0 & 98.7 \end{pmatrix} \text{MHzMHz}^2 \tag{21}$$

$$\sigma^2 = \begin{pmatrix} 39826 & -23902 & -5255 & -23902 & -9535 & 9215 & -5255 & 9215 & -30291 \\ -23902 & 24685 & 2613 & 24685 & 4773 & -9638 & 2613 & -9638 & 19129 \\ -5255 & 2613 & 16091 & 2613 & 7279 & -1970 & 16091 & -1970 & -2024 \\ -23902 & 24685 & 2613 & 24685 & 4773 & -9638 & 2613 & -9638 & 19129 \\ -9535 & 4773 & 7279 & 4773 & 5203 & -2063 & 7279 & -2063 & 4332 \\ 9215 & -9638 & -1970 & -9638 & -2063 & 4734 & -1970 & 4734 & -7152 \\ -5255 & 2613 & 16091 & 2613 & 7279 & -1970 & 16091 & -1970 & -2024 \\ 9215 & -9638 & -1970 & -9638 & -2063 & 4734 & -1970 & 4734 & -7152 \\ -30291 & 19129 & -2024 & 19129 & 4332 & -7152 & -2024 & -7152 & 25959 \end{pmatrix} \text{MHz}^2 \tag{22}$$

12.3. Hyperfine Interactions

For simulations involving multiple spin systems, MD-derived parameters were used for each hyperfine coupling tensor for each atom in the FAD. The first set of coupling tensors were defined in the molecular frame of the FAD. The second set of tensors were created from the first set by transforming each tensor to the eigenframe of the HFC tensor of N5, and choosing the displacement of the superoxide radical to be displaced along x. The molecular frame of FAD is not the same as the eigenframe of N5's HFC, albeit they are similar. The spins are listed here in descending order by magnitude of eigenvalues, as this is an indicator of how much influence each additional spin will exert on the magnetosensitive dynamics. Five spins was the maximum modelled here, due to computational limitations.

12.3.1. In the Molecular Frame of FAD

$$A_{N5} = \begin{pmatrix} 2.091 & -0.133 & 2.274 \\ -0.134 & 1.787 & -0.88 \\ 2.725 & -0.881 & 47.58 \end{pmatrix} \text{MHz} \tag{23}$$

$$A_{N10} = \begin{pmatrix} 1.965 & 0.059 & -3.344 \\ 0.056 & 0.798 & -0.016 \\ -3.343 & -0.016 & 19.941 \end{pmatrix} \text{MHz} \tag{24}$$

$$A_{Hb2} = \begin{pmatrix} 16.053 & -0.031 & -0.757 \\ -0.031 & 12.738 & 0.114 \\ -0.757 & 0.114 & 12.638 \end{pmatrix} \text{MHz} \tag{25}$$

$$A_{H8a} = \begin{pmatrix} 5.911 & 0.522 & -0.11 \\ 0.522 & 6.586 & -0.196 \\ -0.11 & -0.197 & 5.644 \end{pmatrix} \text{MHz} \quad (26)$$

$$A_{Hb1} = \begin{pmatrix} 4.025 & 1.229 & 0.504 \\ 1.227 & 1.861 & 0.235 \\ 0.505 & 0.235 & 1.353 \end{pmatrix} \text{MHz} \quad (27)$$

12.3.2. In the Eigenframe of A_{N5}

Subscripts have been affixed with 'd' to denote a tensor in the eigenframe of A_{N5}

$$A_{N5d} = \begin{pmatrix} -2.6 & 0 & 0 \\ 0 & -2.6 & 0 \\ 0 & 0 & 49.2 \end{pmatrix} \text{MHz} \quad (28)$$

$$A_{N10d} = \begin{pmatrix} 1.017 & 0.577 & 1.342 \\ 0.580 & 2.247 & 4.259 \\ 1.342 & 4.258 & 19.557 \end{pmatrix} \text{MHz} \quad (29)$$

$$A_{Hb2d} = \begin{pmatrix} 13.190 & 1.550 & 0.165 \\ 1.550 & 15.750 & 0.700 \\ 0.165 & 0.700 & 12.567 \end{pmatrix} \text{MHz} \quad (30)$$

$$A_{H8ad} = \begin{pmatrix} 6.857 & 0.359 & 0.235 \\ 0.359 & 5.656 & 0.086 \\ 0.236 & 0.086 & 5.641 \end{pmatrix} \text{MHz} \quad (31)$$

$$A_{Hb1d} = \begin{pmatrix} 3.024 & 1.694 & -0.491 \\ 1.696 & 2.902 & -0.459 \\ -0.492 & -0.460 & 1.399 \end{pmatrix} \text{MHz} \quad (32)$$

Suppl. Note 13: A Perturbation Treatment

We begin with a review of the matrix representation of the effective Hamiltonian shown in the main text. Time-independent degenerate perturbation theory will be applied for further analysis.

Considering a pair of electrons involving exchange and dipole-dipole interactions, where each is coupled to nuclear spins via hyperfine interactions. The spin Hamiltonian has the form

$$\hat{H}(r) = \hat{H}_A + \hat{H}_B + \hat{H}_{AB}(r), \tag{33}$$

where $\hat{H}_A$ and $\hat{H}_B$ are free Hamiltonians of radicals A and B, respectively. $\hat{H}_{AB}(r)$ denotes interactions and depends on the radicals' spatial separation r. The free Hamiltonian is of the form

$$\hat{H}_i = \sum_j \hat{\mathbf{S}}_i \cdot \mathbf{A}_{ij} \cdot \hat{\mathbf{I}}_{ij} - \gamma_i \hat{\mathbf{S}}_i \cdot \mathbf{B}, \tag{34}$$

with $i \in \{A, B\}$. The first term represents hyperfine interactions with coupling tensor $\mathbf{A}$, and the second term arises from the Zeeman interaction with a magnetic field $\mathbf{B}$. In general, spin interactions involve a Heisenberg exchange term, with a scalar coupling J dependent on r, and a electron dipolar term (further details can be found in [15]). Here, we consider four different cases. The first three cases require the dipolar axis of the effective Hamiltonian along the x-direction and an applied magnetic field along the x-, y-, and z-axes. The second one takes the dipolar axis along the y-direction and the applied field along the z-direction.

13.1. Dipolar axis along x-direction

Defining the dipolar axis along x, the electronic interaction Hamiltonian can be written as

$$\begin{aligned}\hat{H}_{AB}(r) =& -J(r)\left(\frac{\hat{1}}{2} + 2\hat{\mathbf{S}}_A \cdot \hat{\mathbf{S}}_B\right) + d(r)(\hat{\mathbf{S}}_A \cdot \hat{\mathbf{S}}_B - 3\hat{S}_A^x \hat{S}_B^x) \\ =& -\frac{\hat{1}}{2}J(r) - \left(\frac{d(r)}{4} + J(r)\right)\left(\hat{S}_A^+\hat{S}_B^- + \hat{S}_A^-\hat{S}_B^+\right) + (d(r) - 2J(r))\hat{S}_A^z\hat{S}_B^z - \frac{3d(r)}{4}\left(\hat{S}_A^+\hat{S}_B^+ + \hat{S}_A^-\hat{S}_B^-\right),\end{aligned} \tag{35}$$

where $d(r) = \mu_0\gamma_A\gamma_B/4\pi(r+R)^3$, and R is the distance of centres of spin density for radicals in contact [15]. Denoting $\hat{S}^\pm = \hat{S}^x \pm \mathrm{i}\hat{S}^y$, the spin Hamiltonian can be given by

$$\begin{aligned}\hat{H}^{\{x,y,z\}}(r) =& \omega_A \hat{S}_A^{\{x,y,z\}} + \omega_B \hat{S}_B^{\{x,y,z\}} + a\hat{S}_A^z - \frac{\hat{1}}{2}J(r) - \left(\frac{d(r)}{4} + J(r)\right)\left(\hat{S}_A^+\hat{S}_B^- + \hat{S}_A^-\hat{S}_B^+\right) \\ &+ (d(r) - 2J(r))\hat{S}_A^z\hat{S}_B^z - \frac{3d(r)}{4}\left(\hat{S}_A^+\hat{S}_B^+ + \hat{S}_A^-\hat{S}_B^-\right),\end{aligned} \tag{36}$$

where ω_i denotes the Larmor precession frequency upon x-, y- or z-component of the applied magnetic field, and an axial hyperfine coupling between one of the electrons and nuclei is considered ($A^{xx} = A^{yy} = 0$, $a \equiv A^{zz}m_I$, and $A^{zz} \gg \omega_i$ for $i \in \{A, B\}$).

We can to express the spin Hamiltonian in the triplet-mixed basis [15],

$$\begin{bmatrix}|T_\Sigma\rangle \\ |T_0\rangle \\ |T_\Delta\rangle \\ |S\rangle\end{bmatrix} \equiv \frac{1}{\sqrt{2}}\begin{bmatrix}|\alpha\alpha\rangle + |\beta\beta\rangle \\ |\alpha\beta\rangle + |\beta\alpha\rangle \\ |\alpha\alpha\rangle - |\beta\beta\rangle \\ |\alpha\beta\rangle - |\beta\alpha\rangle\end{bmatrix}, \tag{37}$$

where their matrix representations are

$$\hat{H}^x_{\text{eff}}(m_I) = \frac{1}{2}\begin{bmatrix} -2J-d & \omega_A+\omega_B+b & a & 0 \\ \omega_A+\omega_B+b & -2J-d & 0 & a \\ a & 0 & -2J+2d & -\omega_A+\omega_B \\ 0 & a & -\omega_A+\omega_B & 2J-\mathrm{i}\kappa \end{bmatrix}, \tag{38}$$

$$\hat{H}^y_{\text{eff}}(m_I) = \frac{1}{2}\begin{bmatrix} -2J-d & b & a & \mathrm{i}(-\omega_A+\omega_B) \\ b & -2J-d & -\mathrm{i}(\omega_A+\omega_B) & a \\ a & \mathrm{i}(\omega_A+\omega_B) & -2J+2d & 0 \\ \mathrm{i}(\omega_A-\omega_B) & a & 0 & 2J-\mathrm{i}\kappa \end{bmatrix}, \tag{39}$$

$$\hat{H}^z_{\text{eff}}(m_I) = \frac{1}{2}\begin{bmatrix} -2J-d & b & \omega_A+\omega_B+a & 0 \\ b & -2J-d & 0 & \omega_A-\omega_B+a \\ \omega_A+\omega_B+a & 0 & -2J+2d & 0 \\ 0 & \omega_A-\omega_B+a & 0 & 2J-\mathrm{i}\kappa \end{bmatrix}. \tag{40}$$

The effective Hamiltonians above include an anti-Hermitian decay term $-\mathrm{i}\kappa/2$ in each singlet state $|S\rangle$ and an additional perturbation, b, in the (1,2) and (2,1) positions.

13.2. Dipolar axis along y-direction

Defining the dipolar axis along y and the applied field direction along z, the spin Hamiltonian can be simplified to

$$\begin{aligned} \hat{H}(r) =& \omega_A\hat{S}^z_A + \omega_B\hat{S}^z_B + a\hat{S}^z_A - \frac{\hat{1}}{2}J(r) - \left(\frac{d(r)}{4} + J(r)\right)\left(\hat{S}^+_A\hat{S}^-_B + \hat{S}^-_A\hat{S}^+_B\right) \\ &+ (d(r) - 2J(r))\hat{S}^z_A\hat{S}^z_B + \frac{3d(r)}{4}\left(\hat{S}^+_A\hat{S}^+_B + \hat{S}^-_A\hat{S}^-_B\right). \end{aligned} \tag{41}$$

The matrix representation of the above Hamiltonian in the triplet-mixed basis reads

$$\hat{H}_{\text{eff}}(m_I) = \frac{1}{2}\begin{bmatrix} -2J+2d & 0 & \omega_A+\omega_B+a & 0 \\ 0 & -2J-d & \mathrm{i}b & \omega_A-\omega_B+a \\ \omega_A+\omega_B+a & -\mathrm{i}b & -2J-d & 0 \\ 0 & \omega_A-\omega_B+a & 0 & 2J-\mathrm{i}\kappa \end{bmatrix}. \tag{42}$$

Here, we have inserted perturbations $\mathrm{i}b$ and $-\mathrm{i}b$ into the (2,3) and (3,2) positions respectively.

13.3. Time-independent Perturbative Expansion

To evaluate the eigenvalues and eigenvectors of the effective Hamiltonian under small perturbations, we begin with the simplest time-independent perturbation theory.

$$\hat{H} = \hat{H}_0 + \epsilon\hat{V} \tag{43}$$

in which the time-independent Schrödinger equation $\hat{H}_0\psi^{(0)}_n = E^{(0)}_n\psi^{(0)}_n$ holds, and eigenfunctions $\psi^{(0)}_n$ form a complete orthonormal set. The eigenvalue problem to be solved is $\hat{H}\psi_n = E_n\psi_n$.

13.3.1. Nondegenerate case

Time-independent perturbation theory expands the eigenvalues and eigenfunctions of $\hat{H}$ in powers of ϵ.

$$\left(\hat{H}_0 + \epsilon\hat{V}\right)\left(\psi^{(0)}_n + \epsilon\psi^{(1)}_n + \epsilon^2\psi^{(2)}_n + \cdots\right) = \left(E^{(0)}_n + \epsilon E^{(1)}_n + \epsilon^2 E^{(2)}_n + \cdots\right)\left(\psi^{(0)}_n + \epsilon\psi^{(1)}_n + \epsilon^2\psi^{(2)}_n + \cdots\right). \tag{44}$$

Collecting powers of ϵ, we have

$$\begin{aligned} \hat{H}_0\psi^{(0)}_n =& E^{(0)}_n\psi^{(0)}_n \\ \hat{H}_0\psi^{(1)}_n + \hat{V}\psi^{(0)}_n =& E^{(0)}_n\psi^{(1)}_n + E^{(1)}_n\psi^{(0)}_n \\ \hat{H}_0\psi^{(2)}_n + \hat{V}\psi^{(1)}_n =& E^{(0)}_n\psi^{(2)}_n + E^{(1)}_n\psi^{(1)}_n + E^{(2)}_n\psi^{(0)}_n \\ &\cdots \end{aligned} \tag{45}$$

We can solve eigenvalues for each order from each line of equation. In short, the approximate energy eigenvalues up to the second order are given by [16]

$$E_n \approx E_n^{(0)} + E_n^{(1)} + E_n^{(2)} = E_n^{(0)} + \langle \psi_n^{(0)} | \hat{V} | \psi_n^{(0)} \rangle + \sum_{k \neq n} \frac{|\langle \psi_n^{(0)} | \hat{V} | \psi_k^{(0)} \rangle|^2}{E_n^{(0)} - E_k^{(0)}}. \tag{46}$$

13.3.2. Degenerate case

We denote $\psi_{nr}^{(0)}$ as wave functions for an α-fold degenerate energy level corresponding to $(r = 1, 2, \ldots, \alpha)$, and we assume the orthonormality [16]

$$\langle \psi_{nr}^{(0)} | \psi_{ns}^{(0)} \rangle = \delta_{rs} \qquad (r, s = 1, 2, \cdots, \alpha). \tag{47}$$

We expand the exact wave function ψ_{nr} in powers of ϵ and introduce a "good" zeroth-order functions $\varphi_{nr}^{(0)}$

$$\psi_{nr} = \varphi_{nr}^{(0)} + \epsilon \psi_{nr}^{(1)} + \epsilon^2 \psi_{nr}^{(2)} + \cdots \tag{48}$$

We notice that as $\epsilon \to 0$, $\psi_{nr} \to \varphi_{nr}^{(0)}$; however, the unperturbed wave functions need not coincide with $\psi_{nr}^{(0)}$. They span the same degenerate subspace and share the same eigenvalues [17]. The perturbed energy E_{nr} can also be expanded as

$$E_{nr} = E_n^{(0)} + \epsilon E_{nr}^{(1)} + \epsilon^2 E_{nr}^{(2)} + \cdots \tag{49}$$

with $E_n^{(0)} = E_{nr}^{(0)}$ $(r = 1, 2, \cdots, \alpha)$. Substituting the above into the Schrödinger equation

$$\left(\hat{H}_0 + \epsilon \hat{V}\right)\left(\varphi_{nr}^{(0)} + \epsilon \psi_{nr}^{(1)} + \epsilon^2 \psi_{nr}^{(2)} + \cdots\right) = \left(E_n^{(0)} + \epsilon E_{nr}^{(1)} + \epsilon^2 E_{nr}^{(2)} + \cdots\right)\left(\varphi_{nr}^{(0)} + \epsilon \psi_{nr}^{(1)} + \epsilon^2 \psi_{nr}^{(2)} + \cdots\right). \tag{50}$$

Likewise, the first-order energy correction can be calculated by hitting the equation of $O(\epsilon)$ with $\langle \psi_{nu}^{(0)}|$ from the left

$$\begin{aligned} \sum_{k,s} a_{nr,ks}^{(1)} (E_k^{(0)} - E_n^{(0)}) \langle \psi_{nu}^{(0)} | \psi_{ks}^{(0)} \rangle + \sum_s c_{rs} [V_{nu,ns} - E_{nr}^{(1)} \delta_{us}] &= 0 \\ \sum_s c_{rs} [V_{nu,ns} - E_{ns}^{(1)}] &= 0, \end{aligned} \tag{51}$$

where we have used the shorthand notation $V_{nu,ns} \equiv \langle \psi_{nu}^{(0)} | \hat{V} | \psi_{ns}^{(0)} \rangle$ and introduced $u = 1, 2, \cdots, \alpha$. The above linear equations with unknown and nontrivial coefficients, $c_{r1}, c_{r2}, \ldots, c_{r\alpha}$, can be solved by the following secular equation

$$\det \left| V_{nu,ns} - E_{nr}^{(1)} \delta_{us} \right| = 0 \tag{52}$$

The first-order correction is given by $E_{nr}^{(1)} = \langle \varphi_{nr}^{(0)} | \hat{V} | \varphi_{ns}^{(0)} \rangle$ and should be diagonalised in this "good" basis, $\varphi_{nr}^{(0)}$. Once the degeneracy can be removed for the first-order, the remaining steps return to the nondegenerate perturbative expansion. We note that the non-Hermiticity of the unperturbed Hamiltonian does not affect the first-order calculations of energy eigenvalues in either the degenerate or non-degenerate cases.

13.4. Expansion for the triplet-mixed basis

13.4.1. Dipolar axis along x-direction

Start with Eq. (38, 39, and 40). All of them have doubly degenerate energies, with a degenerate subspace spanned by $|T_\Sigma\rangle$ and $|T_0\rangle$. We shall first use degenerate perturbation theory to remove the degeneracy and then apply the nondegenerate version up to second order to account for other off-diagonals.

The good basis can be obtained by diagonalising the projection to the degenerate subspace [17].

$$\begin{bmatrix} \frac{1}{\sqrt{2}}(|T_\Sigma\rangle + |T_0\rangle) \\ \frac{1}{\sqrt{2}}(|T_\Sigma\rangle - |T_0\rangle) \\ |T_\Delta\rangle \\ |S\rangle \end{bmatrix} = \begin{bmatrix} \frac{1}{2}(|\alpha\alpha\rangle + |\beta\beta\rangle + |\alpha\beta\rangle + |\beta\alpha\rangle) \\ \frac{1}{2}(|\alpha\alpha\rangle + |\beta\beta\rangle - |\alpha\beta\rangle - |\beta\alpha\rangle) \\ \frac{1}{\sqrt{2}}(|\alpha\alpha\rangle - |\beta\beta\rangle) \\ \frac{1}{\sqrt{2}}(|\alpha\beta\rangle - |\beta\alpha\rangle) \end{bmatrix}. \tag{53}$$

Computing other matrix elements under this basis and combining with the unperturbed non-Hermitian Hamiltonian, our task reduces to tackling the second-order nondegenerate perturbation,

$$\hat{H}^x_{\text{eff}} = \frac{1}{2}\begin{bmatrix} b+\omega_A+\omega_B-d-2J & 0 & \frac{a}{\sqrt{2}} & \frac{a}{\sqrt{2}} \\ 0 & -b-\omega_A-\omega_B-d-2J & \frac{a}{\sqrt{2}} & -\frac{a}{\sqrt{2}} \\ \frac{a}{\sqrt{2}} & \frac{a}{\sqrt{2}} & 2d-2J & -\omega_A+\omega_B \\ \frac{a}{\sqrt{2}} & -\frac{a}{\sqrt{2}} & -\omega_A+\omega_B & 2J-\mathrm{i}\kappa \end{bmatrix}, \tag{54}$$

$$\hat{H}^y_{\text{eff}} = \frac{1}{2}\begin{bmatrix} b-d-2J & 0 & (a-\mathrm{i}(\omega_A+\omega_B))/\sqrt{2} & (a+\mathrm{i}(-\omega_A+\omega_B))/\sqrt{2} \\ 0 & -b-d-2J & (a+\mathrm{i}(\omega_A+\omega_B))/\sqrt{2} & (-a+\mathrm{i}(-\omega_A+\omega_B))/\sqrt{2} \\ (a+\mathrm{i}(\omega_A+\omega_B))/\sqrt{2} & (a-\mathrm{i}(\omega_A+\omega_B))/\sqrt{2} & 2d-2J & 0 \\ (a-\mathrm{i}(-\omega_A+\omega_B))/\sqrt{2} & (-a-\mathrm{i}(\omega_A+\omega_B))/\sqrt{2} & 0 & 2J-\mathrm{i}\kappa \end{bmatrix}, \tag{55}$$

$$\hat{H}^z_{\text{eff}} = \frac{1}{2}\begin{bmatrix} b-d-2J & 0 & (\omega_A+\omega_B+a)/\sqrt{2} & (\omega_A-\omega_B+a)/\sqrt{2} \\ 0 & -b-d-2J & (\omega_A+\omega_B+a)/\sqrt{2} & (-\omega_A+\omega_B-a)/\sqrt{2} \\ (\omega_A+\omega_B+a)/\sqrt{2} & (\omega_A+\omega_B+a)/\sqrt{2} & 2d-2J & 0 \\ (\omega_A-\omega_B+a)/\sqrt{2} & (-\omega_A+\omega_B-a)/\sqrt{2} & 0 & 2J-\mathrm{i}\kappa \end{bmatrix}. \tag{56}$$

Substituting the above Hamiltonians back into Eq. (46), we can get the approximate eigen-energies for each direction of the applied magnetic field

$$\begin{aligned} E^x_1 &= \frac{1}{2}\left[-d+b+\omega_A+\omega_B-2J+\frac{|a|^2}{2(-3d+b+\omega_A+\omega_B)}+\frac{|a|^2}{2(-d+b+\omega_A+\omega_B+\mathrm{i}\kappa-4J)}\right] \\ E^x_2 &= \frac{1}{2}\left[-d-b-(\omega_A+\omega_B)-2J+\frac{|a|^2}{2(-3d-b-(\omega_A+\omega_B))}+\frac{|a|^2}{2(-d-b-(\omega_A+\omega_B)+\mathrm{i}\kappa-4J)}\right] \\ E^x_3 &= \frac{1}{2}\left[2d-2J+\frac{|a|^2}{2(3d-b-(\omega_A+\omega_B))}+\frac{|a|^2}{2(3d+b+(\omega_A+\omega_B))}+\frac{|-\omega_A+\omega_B|^2}{2d+\mathrm{i}\kappa-4J}\right] \\ E^x_4 &= \frac{1}{2}\left[2J-\mathrm{i}\kappa+\frac{|a|^2}{2(4J-\mathrm{i}\kappa+d-b-(\omega_A+\omega_B))}+\frac{|a|^2}{2(4J-\mathrm{i}\kappa+d+b-(\omega_A+\omega_B))}+\frac{|-\omega_A+\omega_B|^2}{4J-\mathrm{i}\kappa-2b}\right], \end{aligned} \tag{57}$$

$$\begin{aligned} E^y_1 &= \frac{1}{2}\left[b-d-2J+\frac{|a-\mathrm{i}(\omega_A+\omega_B)|^2}{2(b-3d)}+\frac{|a+\mathrm{i}(-\omega_A+\omega_B)|^2}{2(b-d+\mathrm{i}\kappa-4J)}\right] \\ E^y_2 &= \frac{1}{2}\left[-b-d-2J+\frac{|a+\mathrm{i}(\omega_A+\omega_B)|^2}{2(-b-3d)}+\frac{|-a+\mathrm{i}(-\omega_A+\omega_B)|^2}{2(-b-d+\mathrm{i}\kappa-4J)}\right] \\ E^y_3 &= \frac{1}{2}\left[2d-2J+\frac{|a+\mathrm{i}(\omega_A+\omega_B)|^2}{2(3d-b)}+\frac{|a-\mathrm{i}(\omega_A+\omega_B)|^2}{2(3d+b)}\right] \\ E^y_4 &= \frac{1}{2}\left[2J-\mathrm{i}\kappa+\frac{|a-\mathrm{i}(-\omega_A+\omega_B)|^2}{2(4J-\mathrm{i}\kappa-b+d)}+\frac{|-a-\mathrm{i}(\omega_A+\omega_B)|^2}{2(4J-\mathrm{i}\kappa+b+d)}\right], \end{aligned} \tag{58}$$

and

$$\begin{aligned} E^z_1 &= \frac{1}{2}\left[b-d-2J+\frac{|\omega_A+\omega_B+a|^2}{2(b-3d)}+\frac{|\omega_A-\omega_B+a|^2}{2(b-d+\mathrm{i}\kappa-4J)}\right] \\ E^z_2 &= \frac{1}{2}\left[-b-d-2J+\frac{|\omega_A+\omega_B+a|^2}{2(-b-3d)}+\frac{|-\omega_A+\omega_B-a|^2}{2(-b-d+\mathrm{i}\kappa-4J)}\right] \\ E^z_3 &= \frac{1}{2}\left[2d-2J+\frac{|\omega_A+\omega_B+a|^2}{2(3d-b)}+\frac{|\omega_A+\omega_B+a|^2}{2(3d+b)}\right] \\ E^z_4 &= \frac{1}{2}\left[2J-\mathrm{i}\kappa+\frac{|\omega_A-\omega_B+a|^2}{2(4J-\mathrm{i}\kappa-b+d)}+\frac{|-\omega_A+\omega_B-a|^2}{2(4J-\mathrm{i}\kappa+b+d)}\right]. \end{aligned} \tag{59}$$

13.4.2. Dipolar axis along y-direction

Begin with the Eq. (42). The good basis that diagonalises the degenerate subspace reads

$$\begin{bmatrix} |T_\Sigma\rangle \\ \frac{1}{\sqrt{2}}(\mathrm{i}\,|T_0\rangle + |T_\Delta\rangle) \\ \frac{1}{\sqrt{2}}(-\mathrm{i}\,|T_0\rangle + |T_\Delta\rangle) \\ |S\rangle \end{bmatrix} = \begin{bmatrix} \frac{1}{\sqrt{2}}(|\alpha\alpha\rangle + |\beta\beta\rangle) \\ \frac{1}{2}(\mathrm{i}\,|\alpha\beta\rangle + \mathrm{i}\,|\beta\alpha\rangle + |\alpha\alpha\rangle - |\beta\beta\rangle) \\ \frac{1}{2}(-\mathrm{i}\,|\alpha\beta\rangle - \mathrm{i}\,|\beta\alpha\rangle + |\alpha\alpha\rangle - |\beta\beta\rangle) \\ \frac{1}{\sqrt{2}}(|\alpha\beta\rangle - |\beta\alpha\rangle) \end{bmatrix}. \tag{60}$$

Now, the Hamiltonian matrix is reduced to

$$\hat{H}_{\mathrm{eff}} = \frac{1}{2}\begin{bmatrix} -2J + 2d & (\omega_A + \omega_B + a)/\sqrt{2} & (\omega_A + \omega_B + a)/\sqrt{2} & 0 \\ (\omega_A + \omega_B + a)/\sqrt{2} & -2J - d + b & 0 & -\mathrm{i}(\omega_A - \omega_B + a)/\sqrt{2} \\ (\omega_A + \omega_B + a)/\sqrt{2} & 0 & -2J - d - b & \mathrm{i}(\omega_A - \omega_B + a)/\sqrt{2} \\ 0 & \mathrm{i}(\omega_A - \omega_B + a)/\sqrt{2} & -\mathrm{i}(\omega_A - \omega_B + a)/\sqrt{2} & 2J - \mathrm{i}k_S \end{bmatrix}. \tag{61}$$

Substituting Eq. (61) back into Eq. (46), we can get the approximate eigen-energies

$$\begin{aligned} E_1 =& \frac{1}{2}\left[2d - 2J + \frac{|\omega_A + \omega_B + a|^2}{2(3d - b)} + \frac{|\omega_A + \omega_B + a|^2}{2(3d + b)}\right] \\ E_2 =& \frac{1}{2}\left[-d + b - 2J + \frac{|\omega_A + \omega_B + a|^2}{2(b - 3d)} + \frac{|\omega_A + \omega_B + a|^2}{2(-d + b + \mathrm{i}\kappa - 4J)}\right] \\ E_3 =& \frac{1}{2}\left[-d - b - 2J + \frac{|\omega_A + \omega_B + a|^2}{2(-3d - b)} + \frac{|\mathrm{i}(\omega_A - \omega_B + a)|^2}{2(-d - b + \mathrm{i}\kappa - 4J)}\right] \\ E_4 =& \frac{1}{2}\left[2J - \mathrm{i}\kappa + \frac{|\mathrm{i}(\omega_A - \omega_B + a)|^2}{2(4J - \mathrm{i}\kappa + d - b)} + \frac{|-\mathrm{i}(\omega_A - \omega_B + a)|^2}{2(4J - \mathrm{i}\kappa + d + b)}\right]. \end{aligned} \tag{62}$$

Suppl. Fig. 15 illustrates a comparison between numerical and approximate energy eigenvalues. We require comparative magnitudes of the dipolar coupling strength d, the hyperfine coupling strength a, and the new perturbation b in a descending order ($d > a > b$). The four cases, using the same set of parameters, lead to identical plots except that the labelling of the solved eigenvalues differs.

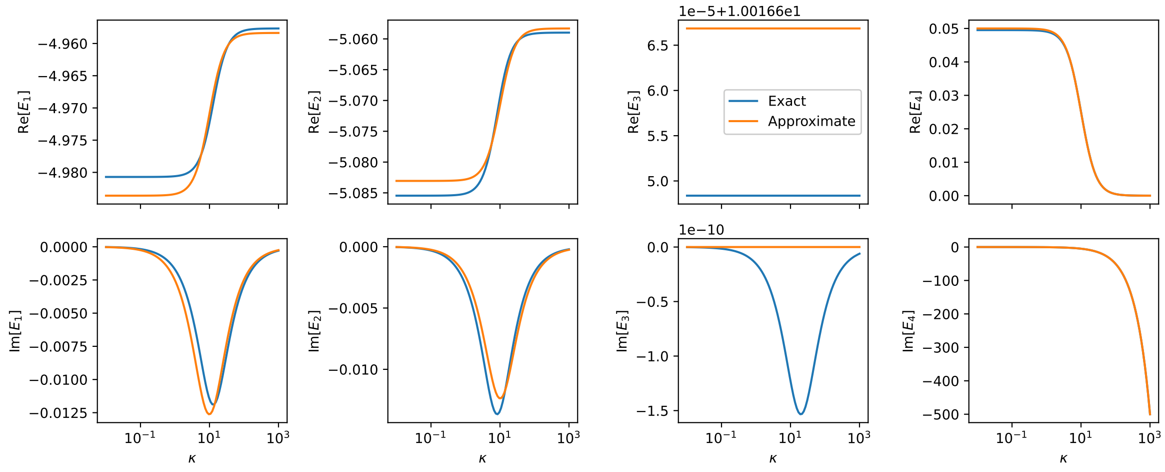

Suppl. Fig. 15: Numerical energy eigenvalues solved by Python's `numpy.linalg.eigvals` and approximate eigenvalues using perturbation theory. In it, $d = 10$, $a = 1$, $b = 0.1$, and $J = \omega_A = \omega_B = 0$ are used.

Suppl. Note 14: Comparison with the Hierarchical Equations of Motion

Here we demonstrate the validity of our Nakajima-Zwanzig approach against the Hierarchical Equations of Motion (HEOM), following the approach taken in [18]. Specifically for the exponential correlation functions $g_{j,k}(t) = \left\langle X_i^* X_j \right\rangle \exp(-t/\tau_C)$ considered in this study, which have the associated spectral densities $\mathcal{J}(\omega) = \left\langle X_i^* X_j \right\rangle (\tau_C^{-1} - \mathrm{i}\omega)^{-1}$, the HEOM takes the form

$$\partial_t \hat{\rho}_{n,n}(t) = (\hat{\hat{\mathcal{L}}}_0 + \hat{\hat{\mathcal{R}}}_0 - n\gamma)\hat{\rho}_{n,n}(t) + \sqrt{(n+1)\langle X^2 \rangle}\hat{\hat{\mathcal{L}}}_1 \hat{\rho}_{n,n+1}(t) + \sqrt{n\langle X^2 \rangle}\hat{\hat{\mathcal{L}}}_1 \hat{\rho}_{n,n-1}(t), \tag{63}$$

where $\gamma = 1/\tau_C$, $\hat{\hat{\mathcal{L}}}_0 = -\mathrm{i}(\hat{H}_{\mathrm{eff}} \cdot - \cdot \hat{H}_{\mathrm{eff}}^\dagger)$ is the Liouvillian of the effective Hamiltonian $\hat{H}_{\mathrm{eff}} = \hat{H} - \mathrm{i}\hat{K}$ as described in the main text, and $\hat{\hat{\mathcal{L}}}_1$ is the Liouvillian describing the, non-Markovian, system-bath interaction. The Markovian part of the system-bath interaction is described by $\hat{\hat{\mathcal{R}}}_0 = S_\delta \hat{\hat{\mathcal{L}}}_1^2$. In the following, we set $S_\delta = 0$ and choose a sufficiently large hierarchy depth, of $n_{\max} = 100$, such that the non-Markovian character of the system-bath interaction is fully accounted for and convergence is achieved.

To assess the validity of our Nakajima-Zwanzig approach, we return to the toy model, described in the main text and Model 1 of the simulation parameters, comprised of a single N5 hyperfine interaction and a dipolar axis along the x-axis. We calculate the compass sensitivity using both the Nakajima-Zwanzig and the HEOM approaches via the anisotropy measure

$$\Delta_S = \Phi_{S,\mathrm{MAX}} - \Phi_{S,\mathrm{MIN}}, \tag{64}$$

where Φ_S is the singlet yield as described in Eq. (9) of the main text. For this toy model this simplifies to assessing the singlet yield in the x-direction and the z-direction.

In Suppl. Fig. 16, we assess the compass sensitivity, Δ_S, against k_S for electron-electron dipolar interaction (EED), exchange interaction (EXR), and axial field relaxation (AFR) where the spin relaxation is along the direction of the magnetic field. We consider system-bath interactions for $\langle X^2 \rangle \tau_C = 0.1\,\mu$s, $\langle X^2 \rangle \tau_C = 1\,\mu s^{-1}$, and $\langle X^2 \rangle \tau_C = 10\,\mu s^{-1}$, and likewise in Suppl. Fig. 17, for which the spin relaxation is considered along the x-axis (xFR). In all cases we see excellent agreement between Nakajima-Zwanzig and HEOM. Thus, for all coupling strengths and correlation times considered in the main text, i.e. in the range $0.1 \leq \langle X^2 \rangle \tau_C \leq 10\,\mu s^{-1}$, we are operating in the valid regime of the Nakajima-Zwanzig approach.

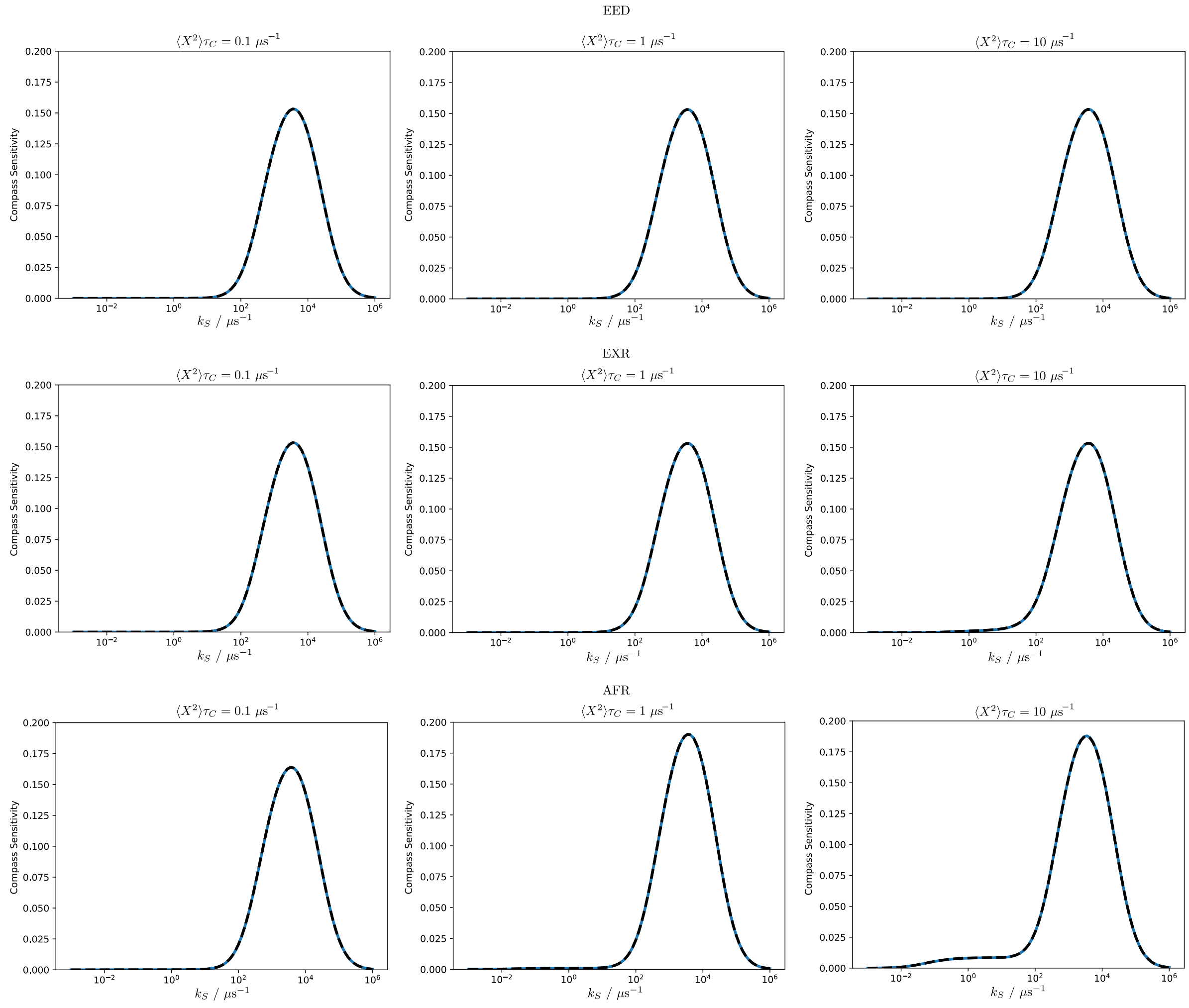

Suppl. Fig. 16: Comparison of compass sensitivity against k_S, with $k_T = 1\,\mu\text{s}^{-1}$, as simulated by Nakajima-Zwanzig (solid lines) and HEOM (dashed lines) with a hierarchy depth of $n_{\text{max}} = 100$, for noise models of axial field relaxation (AFR), electron-electron dipolar interaction (EED), and exchange interaction (EXR), with system-bath coupling of $\langle X^2\rangle\tau_C = 0.1\,\mu s^{-1}$, $\langle X^2\rangle\tau_C = 1\,\mu s^{-1}$ and $\langle X^2\rangle\tau_C = 10\,\mu s^{-1}$.

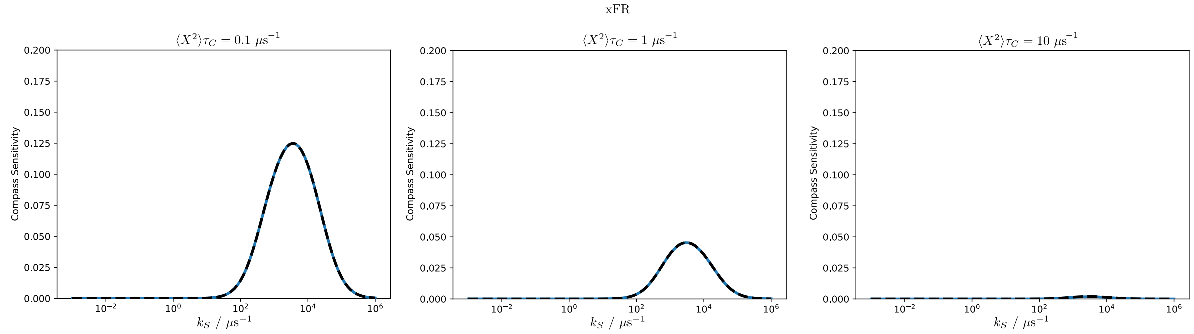

Suppl. Fig. 17: Comparison of compass sensitivity against k_S, with $k_T = 1\,\mu\text{s}^{-1}$, as simulated by Nakajima-Zwanzig (solid lines) and HEOM (dashed lines) with a hierarchy depth of $n_{\text{max}} = 100$, for a noise model of x-field relaxation (xFR), with system-bath coupling of $\langle X^2\rangle\tau_C = 0.1\,\mu\text{s}^{-1}$, $\langle X^2\rangle\tau_C = 1\ \mu\text{s}^{-1}$ and $\langle X^2\rangle\tau_C = 10\,\mu\text{s}^{-1}$.

Supplementary References

1. Cai, J. & Plenio, M. B. Chemical compass model for avian magnetoreception as a quantum coherent device. *Physical review letters* **111,** 230503 (2013).
2. Smith, L. D., Deviers, J. & Kattnig, D. R. Observations about utilitarian coherence in the avian compass. *Scientific reports* **12,** 6011 (2022).
3. Zhang, Y., Berman, G. P. & Kais, S. The radical pair mechanism and the avian chemical compass: Quantum coherence and entanglement. *International Journal of Quantum Chemistry* **115,** 1327–1341 (2015).
4. Gauger, E. M., Rieper, E., Morton, J. J., Benjamin, S. C. & Vedral, V. Sustained quantum coherence and entanglement in the avian compass. *Physical review letters* **106,** 040503 (2011).
5. Katsoprinakis, G., Dellis, A. & Kominis, I. Coherent triplet excitation suppresses the heading error of the avian compass. *New Journal of Physics* **12,** 085016 (2010).
6. Massey, V. Activation of molecular oxygen by flavins and flavoproteins. *J. Biol. Chem.* **269,** 22459–22462 (1994).
7. Toplak, M., Matthews, A. & Teufel, R. The devil is in the details: The chemical basis and mechanistic versatility of flavoprotein monooxygenases. *Archives of Biochemistry and Biophysics* **698,** 108732 (2021).
8. Mladenova, B., Kattnig, D. R. & Grampp, G. ESR-investigations on the dynamic solvent effects of degenerate electron exchange reactions. Part I: Cyanobenzenes. *Zeitschrift für Physikalische Chemie* **220,** 543–562 (2006).
9. Sutin, N. Theory of electron transfer reactions: insights and hindsights. *Progress in Inorganic Chemistry: An Appreciation of Henry Taube,* 441–498 (1983).
10. Stare, J. Oxidation of Flavin by Molecular Oxygen: Computational Insights into a Possible Radical Mechanism. *ACS omega* (2024).
11. Lawan, N., Tinikul, R., Surawatanawong, P., Mulholland, A. J. & Chaiyen, P. QM/MM molecular modeling reveals mechanism insights into flavin peroxide formation in bacterial luciferase. *Journal of Chemical Information and Modeling* **62,** 399–411 (2022).
12. Müller, P. & Ahmad, M. Light-activated cryptochrome reacts with molecular oxygen to form a flavin–superoxide radical pair consistent with magnetoreception. *J. Biol. Chem.* **286,** 21033–21040 (2011).
13. Van Wilderen, L. J., Silkstone, G., Mason, M., van Thor, J. J. & Wilson, M. T. Kinetic studies on the oxidation of semiquinone and hydroquinone forms of Arabidopsis cryptochrome by molecular oxygen. *FEBS Open Bio* **5,** 885–892 (2015).
14. Prabhakar, R., Siegbahn, P. E., Minaev, B. F. & Ågren, H. Activation of triplet dioxygen by glucose oxidase: spin- orbit coupling in the superoxide Ion. *The Journal of Physical Chemistry B* **106,** 3742–3750 (2002).
15. Ramsay, J. L. & Kattnig, D. R. Magnetoreception in cryptochrome enabled by one-dimensional radical motion. *AVS Quantum Sci.,* 022601 (2023).
16. Bransden, B. H. *Quantum mechanics* 375–381 (2000).
17. Sakurai, J. J. & Napolitano, J. *Modern quantum mechanics* 300–303 (2021).
18. Ikeda, T. & Scholes, G. D. Generalization of the hierarchical equations of motion theory for efficient calculations with arbitrary correlation functions. *The Journal of chemical physics* **152** (2020).
