# Supplementary material for: Magnetosensitivity of tightly bound radical pairs in cryptochrome is enabled by the quantum Zeno effect
Source: Nat Commun. 2024 Dec 30;15:10823. doi: 10.1038/s41467-024-55124-x (PMC11686217; doi:10.1038/s41467-024-55124-x)
Supplement: Supplementary file 2 — Description of Additional Supplementary Files [file 41467_2024_55124_MOESM2_ESM.pdf]

Description of Additional Supplementary Files:

Supplementary Data 1: Molecular dynamics geometries.

Initial and final configurations of the molecular dynamics trajectories.
